# Supplementary material for: Induction of pro-inflammatory genes by fibronectin DAMPs in three fibroblast cell lines: Role of TAK1 and MAP kinases
Source: PLoS One. 2023 May 25;18(5):e0286390. doi: 10.1371/journal.pone.0286390 (PMC10212165; doi:10.1371/journal.pone.0286390)
Supplement: S1 Dataset — (PDF) [file pone.0286390.s001.pdf]

Figure1A

Conc (uM)	EDA	III-1c	SEM-1c	SEM-EDA
0.0	0.0	0.0	0.0	0.0
0.5	0.0	0.1	0.0	0.0
1.0	0.1	0.2	0.0	0.0
2.5	0.2	0.5	0.1	0.0
5.0	0.6	1.3	0.2	0.1
7.5	1.1	2.0	0.2	0.1
10.0	1.3	2.3	0.1	0.1
15.0	1.6	2.6	0.1	0.0
20.0	1.7	2.7	0.1	0.0

Figure1C

Conc (uM)	EDA	III-1c	SEM-EDA	SEM-1c
0.0	0.1	0.1	0.0	0.0
0.5	0.1	0.1	0.0	0.0
1.0	0.1	0.2	0.0	0.0
2.5	0.2	0.5	0.0	0.1
5.0	0.4	1.0	0.0	0.2
7.5	0.5	1.4	0.1	0.3
10.0	0.8	1.6	0.1	0.3
15.0	0.9	2.0	0.1	0.3
20.0	1.2	2.3	0.2	0.4

Figure1E

Conc (uM)	EDA	III-1c	SEM-EDA	SEM-1c
0.0	0.0	0.0	0.0	0.0
0.5	0.1	0.0	0.0	0.0
1.0	0.1	0.0	0.0	0.0
2.5	0.3	0.1	0.0	0.0
5.0	0.5	0.2	0.0	0.0
7.5	0.6	0.3	0.0	0.1
10.0	0.6	0.4	0.0	0.1
15.0	0.7	0.6	0.0	0.1
20.0	0.8	0.7	0.0	0.1

Figure1B

Time (hrs)	EDA	III-1C	Add	EDA+III-1C
1	0.0	0.0	0.0	0.0
2	0.1	0.1	0.2	0.9
3	0.2	0.5	0.7	1.8
4	0.5	0.9	1.4	2.7
	SEM-EDA	SEM-1c		SEM-E+c
1	0.0	0.0		0.0
2	0.0	0.0		0.1
3	0.0	0.0		0.2
4	0.1	0.1		0.2

Figure1D

Time (hrs)	EDA	III-1C	Add	EDA+III-1C
1	0.0	0.1	0.1	0.1
2	0.1	0.4	0.5	1.5
3	0.3	0.7	1.0	2.6
4	0.6	1.3	1.8	3.6
	SEM-EDA	SEM-1c		SEM-E+c
1	0.0	0.0		0.0
2	0.0	0.1		0.2
3	0.1	0.3		0.5
4	0.2	0.4		0.3

Figure1F

Time (hrs)	EDA	III-1C	Add	EDA+III-1C
1	0.0	0.0	0.1	0.0
2	0.1	0.1	0.2	0.3
3	0.3	0.2	0.5	0.9
4	0.4	0.4	0.8	1.5
	SEM-EDA	SEM-1c		SEM-E+c
1	0.0	0.0		0.0
2	0.0	0.0		0.1
3	0.0	0.0		0.1
4	0.0	0.1		0.1

Figure2D

AVG	C	FnEDA	FnIII-1C	FnEDA+FnIII-1C
Veh		0.6	0.4	1.0
5O	0.3	0.3	0.3	0.3
5Z		0.6	0.4	1.0

SEM	C	FnEDA	FnIII-1C	FnEDA+FnIII-1C
Veh		0.0	0.0	0.1
5Z	0.0	0.0	0.0	0.0
5O		0.1	0.0	0.1

Figure2E

AVG	C	FnEDA	FnIII-1C	FnEDA+FnIII-1C
Veh		1.1	0.9	1.6
5O	0.5	0.6	0.5	0.6
5Z		1.1	0.9	1.6

SEM	C	FnEDA	FnIII-1C	FnEDA+FnIII-1C
Veh		0.1	0.1	0.1
5Z	0.0	0.1	0.1	0.1
5O		0.1	0.1	0.1

Figure2F

AVG	C	FnEDA	FnIII-1C	FnEDA+FnIII-1C
Veh		0.8	0.6	1.1
5O	0.4	0.5	0.4	0.6
5Z		0.8	0.7	1.1

se=stdev(#)/sqrt(count(#))

SEM	C	FnEDA	FnIII-1C	FnEDA+FnIII-1C
Veh		0.0	0.0	0.1
5Z	0.0	0.0	0.0	0.0
5O		0.0	0.1	0.1

Figure 3A-C

Percent Change		0uM	0.1uM	0.5uM	1uM
EDA	5Z	100	78.5	62.9	89.5
	5O	100	7.3	0.0	0.0
III1c	5Z	100	88.4	75.0	90.0
	5O	100	12.2	0.0	0.0
EDA+III-1c	5Z	100	101.7	96.2	93.7
	5O	100	18.3	0.0	0.0
SEM (in%)					
EDA	5Z	0	0.9	0.2	0.1
	5O	0	9.3	2.6	2.2
III1c	5Z	0	2.0	0.2	0.1
	5O	0	13.1	5.6	9.8
EDA+III-1c	5Z	0	5.2	0.4	0.1
	5O	0	7.1	7.3	7.3

Figure 3D-F

Percent Change		0uM	0.1uM	0.5uM	1uM
EDA	5Z	100	81.5	82.0	65.5
	5O	100	20.8	2.2	0.1
III1c	5Z	100	97.1	92.6	91.5
	5O	100	21.8	1.4	1.0
EDA+III-1c	5Z	100	104.4	98.6	89.2
	5O	100	32.0	0.7	0.5
SEM (in%)					
EDA	5Z	0	2.5	0.3	0.3
	5O	0	2.4	8.4	2.4
III1c	5Z	0	5.1	0.2	0.7
	5O	0	1.7	4.2	1.0
EDA+III-1c	5Z	0	10.2	0.4	0.7
	5O	0	1.6	10.3	18.1

Figure 3G-H

Percent Change		0uM	0.1uM	0.5uM	1uM
EDA	5Z	100	89.1	85.9	86.9
	5O	100	46.7	12.1	4.7
III1c	5Z	100	97.6	101.0	93.4
	5O	100	84.2	21.9	17.0
EDA+III-1c	5Z	100	89.9	84.5	86.4
	5O	100	53.0	7.1	2.5
SEM (in%)					
EDA	5Z	0	2.5	1.2	0.8
	5O	0	4.0	1.4	2.7
III1c	5Z	0	4.0	0.4	0.2
	5O	0	1.8	3.8	1.1
EDA+III-1c	5Z	0	5.1	1.6	0.2
	5O	0	1.9	6.8	7.0

Figure 4A-TAK1 expression

	FnEDA	FnIII-1C	FnEDA+FnIII-1C
-	2.1	2.6	2.3
siCtl	2.2	2.3	2.5
siTAK1	0.3	0.3	0.3
SEM			
-	0.1	0.2	0.1
siCtl	0.2	0.0	0.1
siTAK1	0.1	0.0	0.0

Figure 4C

	FnEDA	FnIII-1C	FnEDA+FnIII-1C
-	2.7	2.4	2.5
siCtl	2.4	2.2	2.4
siTAK1	0.3	0.2	0.2
SEM			
-	0.2	0.1	0.0
siCtl	0.2	0.1	0.1
siTAK1	0.0	0.0	0.0

Figure 4E

	FnEDA	FnIII-1C	FnEDA+FnIII-1C
-	1.5	1.4	1.2
siCtl	1.4	1.4	1.5
siTAK1	0.4	0.4	0.4
SEM			
-	0.0	0.1	0.2
siCtl	0.1	0.1	0.1
siTAK1	0.0	0.0	0.1

Figure 4B-IL8

	FnEDA	FnIII-1C	FnEDA+FnIII-1C
-	0.9	1.5	3.9
siCtl	1.2	1.7	4.3
siTAK1	0.3	0.3	1.8
SEM			
-	0.0	0.1	0.1
siCtl	0.0	0.1	0.1
siTAK1	0.0	0.0	0.2

Figure 4D-IL8

	FnDA	FnIII-1C	FnEDA+FnIII-1C
-	1.5	2.0	5.7
siCtl	1.3	1.7	5.0
siTAK1	0.1	0.1	1.4
SEM			
-	0.1	0.2	0.1
siCtl	0.2	0.2	0.1
siTAK1	0.0	0.0	0.4

Figure 4F-IL8

	FnEDA	FnIII-1C	FnEDA+FnIII-1C
-	1.2	0.7	2.1
siCtl	1.3	1.0	2.3
siTAK1	0.4	0.2	1.1
SEM			
-	0.1	0.1	0.1
siCtl	0.1	0.1	0.1
siTAK1	0.1	0.0	0.0

FIGURE 5

A1F

**pIKK**	FnEDA	FnIII-1C	FnEDA+FnIII-1C	SEM-EDA	SEM-1c	SEM-E+c
Ctl	0.04	0.04	0.04	0.00	0.00	0.00
DMSO	0.09	0.08	0.27	0.00	0.00	0.01
5O	0.04	0.04	0.07	0.00	0.01	0.01
5Z	0.10	0.08	0.27	0.00	0.01	0.02

**pNFkB**	FnEDA	FnIII-1C	FnEDA+FnIII-1C	SEM-EDA	SEM-1c	SEM-E+c
Ctl	0.56	0.56	0.56	0.15	0.15	0.15
DMSO	2.83	1.98	3.23	0.39	0.23	0.17
5O	1.13	0.62	1.16	0.33	0.15	0.13
5Z	2.80	1.64	3.61	0.42	0.29	0.19

**pERK**	FnEDA	FnIII-1C	FnEDA+FnIII-1C	SEM-EDA	SEM-1c	SEM-E+c
Ctl	0.34	0.34	0.34	0.01	0.01	0.01
DMSO	0.98	0.82	2.28	0.04	0.06	0.12
5O	0.23	0.22	0.14	0.02	0.02	0.01
5Z	1.03	0.87	2.11	0.04	0.07	0.09

**pJNK**	FnEDA	FnIII-1C	FnEDA+FnIII-1C	SEM-EDA	SEM-1c	SEM-E+c
Ctl	0.07	0.07	0.07	0.00	0.00	0.00
DMSO	0.27	0.23	0.95	0.01	0.00	0.09
5O	0.07	0.09	0.18	0.00	0.00	0.02
5Z	0.28	0.24	0.98	0.02	0.01	0.13

**pP38**	FnEDA	FnIII-1C	FnEDA+FnIII-1C	SEM-EDA	SEM-1c	SEM-E+c
Ctl	0.10	0.10	0.10	0.00	0.00	0.00
DMSO	1.65	1.19	2.44	0.01	0.04	0.07
5O	0.03	0.04	0.11	0.00	0.00	0.00
5Z	1.61	1.20	2.42	0.02	0.04	0.14

FIGURE 6

HDF

**pIKK**	FnEDA	FnIII-1C	FnEDA+FnIII-1C	SEM-EDA	SEM-1c	SEM-E+c
Ctl	0.02	0.02	0.02	0.00	0.00	0.00
DMSO	0.06	0.06	0.17	0.00	0.00	0.01
5O	0.03	0.03	0.04	0.00	0.00	0.00
5Z	0.06	0.06	0.17	0.00	0.00	0.01

**pNFkB**	FnEDA	FnIII-1C	FnEDA+FnIII-1C	SEM-EDA	SEM-1c	SEM-E+c
Ctl	0.36	0.36	0.36	0.02	0.01	0.01
DMSO	1.88	1.46	4.23	0.08	0.08	0.51
5O	0.37	0.56	1.65	0.02	0.10	0.29
5Z	1.89	1.55	5.03	0.12	0.09	0.92

**pERK**	FnEDA	FnIII-1C	FnEDA+FnIII-1C	SEM-EDA	SEM-1c	SEM-E+c
Ctl	0.34	0.64	0.64	0.01	0.01	0.01
DMSO	0.98	1.14	2.41	0.02	0.05	0.27
5O	0.23	0.33	0.33	0.02	0.06	0.04
5Z	1.03	1.27	2.45	0.08	0.15	0.24

**pJNK**	FnEDA	FnIII-1C	FnEDA+FnIII-1C	SEM-EDA	SEM-1c	SEM-E+c
Ctl	0.08	0.08	0.08	0.00	0.00	0.00
DMSO	0.12	0.11	0.27	0.00	0.01	0.00
5O	0.07	0.06	0.12	0.01	0.00	0.01
5Z	0.11	0.12	0.25	0.01	0.01	0.02

**pP38**	FnEDA	FnIII-1C	FnEDA+FnIII-1C	SEM-EDA	SEM-1c	SEM-E+c
Ctl	0.34	0.34	0.34	0.01	0.01	0.01
DMSO	1.34	1.43	4.82	0.13	0.24	0.83
5O	0.25	0.23	0.38	0.03	0.03	0.15
5Z	1.63	1.41	4.84	0.19	0.30	0.48

FIGURE 7

HKF

**pIKK**	FnEDA	FnIII-1C	FnEDA+FnIII-1C	SEM-EDA	SEM-1c	SEM-E+c
Ctl	0.06	0.06	0.06	0.00	0.00	0.00
DMSO	0.13	0.10	0.15	0.01	0.00	0.00
5O	0.07	0.06	0.08	0.00	0.01	0.00
5Z	0.12	0.09	0.16	0.00	0.01	0.00

**pNFkB**	FnEDA	FnIII-1C	FnEDA+FnIII-1C	SEM-EDA	SEM-1c	SEM-E+c
Ctl	0.53	0.53	0.53	0.03	0.03	0.03
DMSO	1.21	0.67	1.70	0.05	0.03	0.13
5O	0.78	0.44	1.06	0.04	0.02	0.07
5Z	1.24	0.65	1.87	0.09	0.02	0.07

**pERK**	FnEDA	FnIII-1C	FnEDA+FnIII-1C	SEM-EDA	SEM-1c	SEM-E+c
Ctl	0.26	0.26	0.26	0.02	0.02	0.02
DMSO	0.56	0.37	0.72	0.02	0.01	0.04
5O	0.31	0.24	0.26	0.01	0.01	0.02
5Z	0.53	0.36	0.72	0.03	0.02	0.05

**pJNK**	FnEDA	FnIII-1C	FnEDA+FnIII-1C	SEM-EDA	SEM-1c	SEM-E+c
Ctl	0.13	0.13	0.13	0.01	0.01	0.01
DMSO	0.19	0.19	0.28	0.01	0.01	0.02
5O	0.13	0.12	0.19	0.01	0.01	0.02
5Z	0.21	0.20	0.29	0.01	0.02	0.03

**pP38**	FnEDA	FnIII-1C	FnEDA+FnIII-1C	SEM-EDA	SEM-1c	SEM-E+c
Ctl	0.15	0.15	0.15	0.01	0.01	0.01
DMSO	0.32	0.27	0.99	0.02	0.01	0.17
5O	0.13	0.11	0.36	0.01	0.01	0.02
5Z	0.35	0.28	1.04	0.01	0.03	0.10

FIGURE 8-A1F

Position		NT Control Group	EDA Group 1	EDA+TAK1i Group 2	EDA+P38i Group 3	EDA+ERKi Group 4	EDA+JNKi Group 5	III-1C Group 6	III-1C+TAK1i Group 7	III-1C+P38i Group 8	III-1C+ERKi Group 9	III-1C+JNKi Group 10
A01	BCL6	28.66	28.13	28.56	30.34	30.58	30.09	27.24	27.98	29.76	30.07	29.18
A02	C3	29.45	28.22	29.51	31.65	32.17	31.03	27.71	29.05	31.51	30.79	31.34
A03	C3AR1	34.31	33.81	34.43	36.37	35.57	36.26	32.85	33.80	35.26	34.03	34.70
A04	CCL11	33.63	29.71	33.77	35.00	34.59	35.14	29.76	33.66	33.69	32.69	34.25
A05	CCL13	35.49	33.51	35.37	35.91	36.89	35.60	33.37	35.36	35.65	35.74	34.57
A06	CCL16	0.00	0.00	0.00	0.00	0.00	0.00	0.00	0.00	0.00	0.00	0.00
A07	CCL17	37.80	37.34	36.88	38.74	37.97	38.51	38.17	35.43	37.11	38.24	36.68
A08	CCL19	34.81	34.72	34.82	38.72	36.74	38.59	34.43	34.63	38.50	37.56	37.07
A09	CCL2	30.59	24.99	32.73	27.78	28.32	28.57	24.83	32.93	26.96	26.13	27.49
A10	CCL21	35.43	36.15	34.94	36.76	36.81	36.13	35.96	34.49	37.96	38.36	36.30
A11	CCL22	0.00	0.00	0.00	0.00	0.00	0.00	0.00	0.00	0.00	0.00	0.00
A12	CCL23	0.00	0.00	0.00	0.00	0.00	0.00	0.00	0.00	0.00	0.00	0.00
B01	CCL24	39.47	0.00	39.67	39.62	0.00	0.00	38.17	38.92	0.00	0.00	0.00
B02	CCL3	39.83	39.17	38.37	0.00	0.00	0.00	39.03	38.10	39.20	0.00	38.16
B03	CCL4	0.00	0.00	0.00	38.67	0.00	0.00	0.00	38.10	0.00	0.00	0.00
B04	CCL5	35.81	33.38	38.56	35.91	37.86	39.84	34.52	34.94	35.18	37.41	38.49
B05	CCL7	32.05	28.13	33.53	29.89	31.08	32.23	28.06	33.21	30.90	30.17	32.57
B06	CCL8	38.87	38.12	37.42	38.10	37.12	37.53	37.02	37.13	38.53	36.83	37.15
B07	CCR1	35.01	35.10	34.33	37.53	36.10	33.99	34.30	34.00	35.77	36.29	34.71
B08	CCR2	32.23	33.97	34.20	34.62	33.05	35.35	32.53	32.94	34.91	33.32	35.14
B09	CCR3	35.73	36.27	36.68	36.96	36.41	35.82	34.99	34.79	35.95	36.19	34.78
B10	CCR4	36.13	37.01	37.41	37.92	37.13	38.59	36.42	36.55	37.02	0.00	35.33
B11	CCR7	31.44	32.25	31.22	36.08	35.12	35.76	31.75	31.16	35.69	34.71	35.76
B12	CD14	30.66	31.00	30.81	32.37	32.43	32.19	30.32	30.71	31.47	31.23	31.05
C01	CD40	27.85	27.85	27.77	29.88	30.25	29.76	27.07	27.65	29.43	29.13	29.18
C02	CD40LG	34.55	37.34	35.44	35.77	35.39	35.58	36.37	33.67	35.19	35.09	35.16
C03	CEBPB	25.67	25.70	25.73	27.34	27.94	27.39	24.91	25.53	27.24	26.56	26.56
C04	CRP	0.00	0.00	0.00	0.00	0.00	0.00	0.00	39.88	0.00	39.95	0.00
C05	CSF1	26.91	25.63	27.11	27.63	27.92	27.92	25.13	26.89	27.23	27.19	27.54
C06	CXCL1	30.93	23.36	33.59	24.43	25.35	26.81	23.11	34.18	24.68	24.73	27.39
C07	CXCL10	38.56	32.35	39.30	36.24	37.32	39.48	33.30	37.55	39.71	34.60	39.17
C08	CXCL2	31.48	24.05	33.56	25.22	25.76	27.25	23.88	33.52	25.26	25.12	27.51
C09	CXCL3	33.41	26.44	33.21	27.64	27.76	29.31	26.48	34.02	27.76	27.67	29.41
C10	CXCL5	32.89	30.28	33.01	30.87	31.17	31.83	30.22	32.43	31.49	31.61	32.73
C11	CXCL6	32.64	28.07	32.23	29.40	30.01	30.13	28.31	32.09	31.40	31.33	32.52
C12	CXCL9	33.60	33.60	34.33	34.14	34.50	33.82	33.71	33.32	34.14	33.74	33.79
D01	CXCR1	0.00	0.00	0.00	0.00	0.00	0.00	0.00	0.00	0.00	0.00	0.00
D02	CXCR2	31.93	31.96	32.04	35.11	32.45	32.30	31.29	31.19	32.24	32.01	31.50
D03	CXCR4	37.39	0.00	0.00	35.88	0.00	0.00	0.00	0.00	0.00	0.00	37.09
D04	FASLG	36.70	36.64	36.53	37.74	37.04	36.39	36.04	35.32	37.08	36.40	35.55
D05	FOS	33.20	33.26	34.34	30.01	34.69	34.03	32.03	34.50	33.30	34.76	34.42
D06	IFNG	39.13	38.55	39.35	0.00	37.11	37.58	38.55	36.52	37.43	37.31	37.01
D07	IL10	0.00	38.27	36.83	0.00	38.17	38.27	37.87	35.82	38.12	39.16	38.15
D08	IL10RB	26.50	26.39	26.34	27.80	27.90	27.91	25.86	26.19	27.49	27.08	27.12

A

D09	IL15	29.04	28.96	28.63	30.51	30.57	31.02	28.45	28.41	30.59	29.99	30.33
D10	IL17A	0.00	0.00	0.00	0.00	0.00	0.00	38.39	0.00	0.00	0.00	0.00
D11	IL18	34.39	34.71	34.04	36.47	35.44	35.48	33.80	33.36	35.32	35.87	33.97
D12	IL1A	30.69	30.17	31.59	32.08	32.28	33.26	29.61	31.84	32.32	31.54	33.32
E01	IL1B	27.84	26.85	28.73	28.36	29.79	31.18	26.19	29.05	28.57	28.25	31.45
E02	IL1R1	25.41	25.78	26.03	26.44	26.33	26.24	24.64	25.72	26.90	26.44	26.12
E03	IL1RAP	30.02	29.94	29.94	31.03	31.31	31.12	29.24	29.92	31.09	31.01	31.23
E04	IL1RN	27.67	27.39	27.76	28.79	27.99	28.54	26.85	27.49	29.41	28.25	28.85
E05	IL22	19.65	34.15	35.08	34.96	34.92	34.55	33.90	34.16	34.61	34.59	34.33
E06	IL23A	31.10	30.97	30.49	33.36	33.75	33.25	30.13	30.34	33.47	33.52	32.97
E07	IL23R	0.00	0.00	38.34	0.00	0.00	0.00	0.00	0.00	0.00	0.00	0.00
E08	IL5	37.45	34.68	35.09	35.72	37.56	37.18	34.98	0.00	37.17	38.33	36.97
E09	IL6	34.64	29.70	35.26	31.81	29.97	33.32	30.04	34.18	31.34	30.07	34.00
E10	IL6R	31.26	32.20	30.81	32.42	32.28	32.67	30.62	30.48	33.02	32.40	31.95
E11	CXCL8	32.35	25.58	33.18	25.51	26.98	29.75	25.22	31.97	26.14	26.47	31.14
E12	IL9	39.93	39.36	39.60	39.15	0.00	0.00	37.92	39.23	38.11	39.20	38.16
F01	ITGB2	34.29	34.33	35.35	39.52	36.12	35.93	33.71	33.39	35.58	37.49	36.66
F02	KNG1	0.00	37.32	37.08	0.00	0.00	0.00	38.01	0.00	0.00	38.87	0.00
F03	LTA	33.06	33.42	33.62	35.10	33.99	34.09	32.35	32.27	34.76	33.69	33.37
F04	LTB	34.23	33.55	34.27	36.90	35.24	34.47	32.58	32.69	34.58	34.13	33.68
F05	LY96	26.51	26.87	26.58	27.06	27.06	27.14	26.17	26.28	27.25	27.04	26.86
F06	MYD88	28.13	28.08	27.94	29.23	29.48	29.26	27.51	27.97	29.52	29.01	29.14
F07	NFKB1	27.63	26.86	27.88	29.00	28.93	29.35	26.47	27.77	28.58	27.90	28.75
F08	NOS2	35.32	35.71	36.24	36.26	39.06	39.75	35.17	34.21	36.09	36.72	36.16
F09	NR3C1	25.64	25.97	25.69	27.64	27.80	27.76	25.32	25.59	27.46	26.94	27.17
F10	PTGS2	30.01	27.68	30.82	29.31	28.37	30.57	26.99	30.78	29.37	28.01	30.29
F11	RIPK2	27.94	27.00	27.75	28.12	28.26	28.38	26.68	27.70	28.24	27.87	28.33
F12	SELE	36.13	37.02	38.08	38.26	37.20	36.96	35.65	36.91	37.25	38.51	36.03
G01	TIRAP	29.62	29.66	29.79	31.73	32.13	31.37	29.04	29.69	31.94	31.15	31.21
G02	TLR1	34.71	35.81	35.62	38.75	0.00	37.94	34.42	35.11	37.06	0.00	36.86
G03	TLR2	32.64	31.64	33.49	33.38	34.11	34.15	31.03	33.04	33.30	33.73	35.34
G04	TLR3	31.40	30.76	31.13	34.27	34.30	35.85	30.75	31.20	33.27	32.86	33.60
G05	TLR4	28.83	28.48	29.50	30.18	31.20	30.45	27.41	29.07	30.26	30.73	30.39
G06	TLR5	34.80	33.20	33.67	37.06	37.88	37.40	33.33	34.24	35.36	35.08	37.31
G07	TLR6	30.06	29.85	30.35	30.86	30.57	30.43	29.20	29.60	31.22	31.25	31.08
G08	TLR7	36.55	34.70	35.78	35.01	35.65	34.58	34.17	35.55	36.17	36.10	35.12
G09	TLR9	34.05	33.97	35.14	35.26	37.19	0.00	35.55	33.70	36.80	39.43	38.27
G10	TNF	37.30	31.19	0.00	33.38	34.31	34.57	31.14	0.00	32.27	32.03	33.50
G11	TNFSF14	36.75	36.52	37.69	38.46	37.43	38.86	36.16	36.32	37.43	0.00	36.69
G12	TOLLIP	26.75	26.73	26.72	28.17	28.38	28.28	26.07	26.63	28.23	27.65	28.03
H01	ACTB	19.16	19.43	19.06	21.15	21.52	21.09	18.65	18.93	20.73	20.15	20.14
H02	B2M	21.44	21.40	21.17	22.23	22.24	22.02	20.73	21.16	22.52	21.95	21.97
H03	GAPDH	20.23	20.70	20.31	22.11	22.23	21.88	20.01	20.23	21.30	20.78	20.97
H04	HPRT1	27.67	27.92	27.38	28.45	28.40	28.22	27.19	27.43	28.41	28.04	28.05
H05	RPLP0	19.28	19.78	19.40	20.10	20.26	20.08	19.01	19.21	20.21	19.66	19.81
H06		39.94	0.00	0.00	37.17	38.79	0.00	39.61	38.02	0.00	0.00	37.72
H07		25.24	25.85	25.55	24.44	24.29	24.29	25.17	25.29	24.50	24.28	24.32

H08	25.22	25.78	25.77	24.39	24.37	24.33	25.24	25.27	24.40	24.07	24.08
H09	25.20	25.76	25.63	24.29	24.28	24.34	25.19	25.20	24.28	24.18	24.19
H10	20.53	20.67	20.57	20.91	20.74	20.92	20.35	20.38	20.57	20.61	20.47
H11	20.50	20.72	20.60	20.99	20.78	20.98	20.36	20.35	20.94	20.53	20.50
H12	20.65	21.03	20.78	21.15	21.11	21.09	20.52	20.54	21.19	20.64	20.74

FIGURE 8-HDF

Position		NT Control Group	EDA Group 1	EDA+TAK1i Group 2	EDA+P38i Group 3	EDA+ERKi Group 4	EDA+JNKi Group 5	III-1C Group 6	III-1C+TAK1i Group 7	III-1C+P38i Group 8	III-1C+ERKi Group 9	III-1C+JNKi Group 10
A01	BCL6	26.35	27.09	27.41	27.01	26.94	26.42	26.59	26.33	26.49	26.56	26.26
A02	C3	28.58	28.34	29.23	28.50	28.44	28.69	28.77	28.65	28.39	28.63	28.88
A03	C3AR1	35.16	34.87	34.96	34.22	35.06	33.56	34.33	35.62	35.03	34.76	35.03
A04	CCL11	32.55	30.06	33.45	31.34	30.45	32.00	31.02	33.29	31.79	31.18	32.64
A05	CCL13	35.76	35.29	36.10	36.60	35.90	37.18	36.11	33.83	36.35	36.59	37.37
A06	CCL16		39.16		39.03							
A07	CCL17	36.77	37.25	37.28	37.05	35.92	35.65	37.95	37.98	35.81	35.76	36.15
A08	CCL19	37.72	36.52	36.09	36.98	38.17	36.43	36.04	35.35	36.64	36.35	36.04
A09	CCL2	31.62	26.22	35.12	27.92	27.36	28.43	26.59	33.88	29.03	27.39	30.80
A10	CCL21	36.40	37.33	36.69	37.80	36.88	36.23	36.31	36.96	37.69	36.59	36.52
A11	CCL22	38.38					37.49				38.76	
A12	CCL23	37.53				38.34	38.05	37.53	39.41			
B01	CCL24				37.65		38.97			38.44		
B02	CCL3	39.21	38.50		38.09	38.07	38.28	38.63		38.18	38.33	38.86
B03	CCL4				35.25	36.51	38.39	37.29		36.20		
B04	CCL5	35.63	31.55	35.43	31.60	32.63	33.14	33.18	34.74	32.05	33.31	36.16
B05	CCL7	33.06	29.19	33.59	29.32	31.36	32.61	30.00	33.29	30.30	31.68	33.14
B06	CCL8	37.40	36.49	38.54	37.58	37.56	38.40	36.20	37.33	37.39	38.06	38.79
B07	CCR1	33.63	32.09	33.23	32.38	33.45	32.74	32.41	33.12	33.09	33.97	32.34
B08	CCR2	34.62	34.46	34.88	35.05	34.52	32.25	34.73	34.78	35.39	35.27	35.04
B09	CCR3	35.80	34.65	34.94	35.06	35.47	35.67	36.01	34.89	36.46	38.00	36.20
B10	CCR4	36.99	37.13	37.41	36.29	37.72	36.17	37.56	36.23	38.93	37.23	36.89
B11	CCR7	31.81	31.43	31.84	32.94	31.66	34.13	32.05	32.26	33.58	31.79	34.56
B12	CD14	29.05	29.02	29.04	29.07	29.14	29.19	29.02	28.40	28.92	29.05	28.89
C01	CD40	30.13	30.03	30.42	30.08	30.13	30.17	30.33	30.04	30.16	30.17	30.47
C02	CD40LG	34.38	35.32	35.13	35.04	35.15	34.76	35.88	37.67	34.76	34.99	35.49
C03	CEBPB	25.02	24.70	25.87	25.29	24.99	24.45	24.36	24.87	25.03	24.67	24.68
C04	CRP		36.66	37.83	38.65				37.47	37.03	37.75	37.96
C05	CSF1	25.40	24.74	26.08	24.81	24.75	25.07	24.88	25.55	24.73	24.77	25.48
C06	CXCL1	29.39	22.41	33.35	22.36	23.29	24.98	22.83	34.03	22.49	23.60	27.65
C07	CXCL10	37.32	31.03		33.23	32.06	35.65	31.89		33.98	33.10	36.51
C08	CXCL2	30.15	22.78	32.04	22.80	23.39	25.17	23.17	33.02	23.14	23.80	27.24
C09	CXCL3	31.60	24.54	31.27	24.42	25.11	25.86	25.03	33.10	24.45	25.58	27.71
C10	CXCL5	30.39	30.14	31.02	30.07	30.15	30.16	30.12	30.32	30.15	30.16	30.38
C11	CXCL6	27.85	26.33	28.20	26.82	27.25	27.68	26.69	27.45	27.01	27.20	27.75
C12	CXCL9	33.34	33.64	34.33	34.68	34.43	33.23	33.96	33.63	33.62	33.39	32.60
D01	CXCR1								39.24	35.27		

D02	CXCR2	31.92	32.17	32.24	32.33	32.64	32.29	32.45	31.22	32.24	32.37	32.42
D03	CXCR4	37.62	37.34						36.88	39.54		37.71
D04	FASLG	36.52	36.30	36.62	35.82	36.97	37.00	36.79	35.64	36.03	37.24	36.36
D05	FOS	31.61	31.30	32.61	28.95	32.20	31.65	30.86	32.31	28.94	32.08	31.27
D06	IFNG	38.70	39.99	37.56	38.42	37.64	37.32	38.15	36.68	37.32	37.47	38.25
D07	IL10	37.22	39.41	37.51	36.08	37.90	38.08	36.41	36.69	37.10	37.01	36.60
D08	IL10RB	26.27	25.96	26.34	25.88	26.14	26.22	26.12	25.88	26.17	26.24	26.32
D09	IL15	28.81	28.23	28.57	28.26	28.49	28.86	28.44	27.90	28.62	28.62	28.62
D10	IL17A						37.80	37.36	39.10		37.82	
D11	IL18	35.13	33.92	34.68	34.27	34.63	34.35	34.12	34.33	34.38	35.27	35.23
D12	IL1A	34.99	33.46	36.77	34.26	34.33	35.58	34.14	34.61	35.19	34.23	34.16
E01	IL1B	36.43	32.05	37.35	31.07	34.21	36.82	33.58	37.99	32.36	34.89	38.90
E02	IL1R1	23.60	23.93	24.13	24.10	23.90	23.87	23.99	23.67	24.00	23.91	24.06
E03	IL1RAP	28.50	28.67	29.00	28.65	28.47	28.34	28.94	28.26	28.36	28.70	28.37
E04	IL1RN	29.50	29.34	29.61	30.48	28.85	30.19	29.76	29.31	30.05	29.11	30.48
E05	IL22	34.16	34.94	34.77	34.57	34.10	33.81	34.42	34.37	34.59	34.20	35.17
E06	IL23A	31.93	32.08	32.65	32.05	32.04	31.95	32.27	31.46	31.68	31.98	32.07
E07	IL23R		38.25								37.96	
E08	IL5	35.45	37.61	36.34	36.29	35.49	36.01	36.43	35.58	37.31	38.33	36.54
E09	IL6	34.32	26.56	34.79	27.52	27.68	30.66	28.00	34.60	28.33	28.78	32.70
E10	IL6R	29.61	30.50	30.14	29.67	30.14	29.99	30.14	29.68	29.54	30.15	29.53
E11	CXCL8	29.91	21.88	32.16	21.99	24.02	26.03	22.32	31.70	22.21	24.18	28.31
E12	IL9	36.51	39.29	39.28	38.12	39.39					38.40	
F01	ITGB2	31.61	31.45	32.03	31.29	31.70	32.07	31.51	31.13	31.96	31.68	31.46
F02	KNG1		39.05		38.66	39.87				39.10	39.26	
F03	LTA	33.33	34.29	33.93	33.73	34.17	33.76	34.08	34.06	33.82	34.86	34.29
F04	LTB	33.36	33.75	33.64	34.91	33.98	33.38	34.13	33.88	33.61	34.55	34.04
F05	LY96	27.07	27.16	27.12	26.94	27.26	27.14	27.11	26.66	27.13	27.22	27.09
F06	MYD88	27.87	28.08	28.17	28.00	28.26	28.29	27.98	27.37	28.20	28.17	28.05
F07	NFKB1	27.62	25.93	27.78	26.10	26.49	27.16	26.15	27.05	26.27	26.66	27.12
F08	NOS2	35.92	35.10	35.44	34.77	34.99	36.33	36.12	34.52	35.05	35.42	35.29
F09	NR3C1	24.96	24.73	25.15	25.18	25.26	25.40	24.78	24.56	25.27	25.39	25.14
F10	PTGS2	28.84	26.46	31.75	27.38	27.44	30.15	26.24	30.52	27.62	27.29	29.49
F11	RIPK2	27.49	25.42	27.65	25.96	26.67	26.48	26.00	27.30	26.31	26.74	26.71
F12	SELE	36.81	37.25	38.55	35.01	37.52		37.78	36.24	36.94	37.16	37.35
G01	TIRAP	30.33	30.79	30.91	30.47	30.95	30.75	30.86	30.16	30.51	30.83	30.57
G02	TLR1	34.68	35.06	35.17	34.87	34.79	34.79	34.58	34.58	35.17	34.56	34.48
G03	TLR2	32.74	30.62	32.98	30.76	32.36	32.10	30.94	33.13	30.43	32.13	32.87
G04	TLR3	31.49	31.47	31.49	31.82	30.99	31.80	31.42	31.00	31.40	31.25	31.14
G05	TLR4	28.04	28.15	29.09	28.11	29.09	28.58	28.08	28.44	28.04	28.87	28.60

G06	TLR5	35.17	36.96	35.38	35.68	35.03	35.92	36.01	36.10	35.05	36.09	37.02
G07	TLR6	29.54	30.26	30.00	30.20	30.03	30.17	29.94	29.96	29.98	30.37	30.35
G08	TLR7	35.27	35.85	36.06	34.69	35.57	35.03	35.70	34.42	34.74	34.93	35.35
G09	TLR9	34.04	34.96	34.87	34.46	35.85	35.28	34.99	34.07	34.67	35.31	36.34
G10	TNF	35.50	30.95	38.52	31.64	31.75	31.72	31.71	38.51	32.01	32.25	33.22
G11	TNFSF14	37.69	37.81	38.95	36.17	37.38	37.04	37.64	37.70	37.25	39.07	37.75
G12	TOLLIP	26.44	26.71	27.28	26.63	26.44	26.57	26.62	27.09	26.52	26.50	26.72
H01	ACTB	19.65	19.60	19.89	19.73	19.97	19.97	20.32	19.21	19.90	19.96	19.66
H02	B2M	20.56	20.35	20.56	20.49	20.61	20.60	20.46	20.06	20.58	20.60	20.39
H03	GAPDH	20.13	20.09	20.22	20.10	20.29	20.17	20.17	19.66	21.21	20.31	20.11
H04	HPRT1	28.14	28.06	28.00	28.00	28.22	28.30	28.06	27.86	28.31	28.11	28.08
H05	RPLP0	19.39	19.36	19.38	19.36	19.66	19.41	19.58	19.09	19.66	19.72	19.43
H06				39.05	37.70		36.63			37.80		38.71
H07		24.02	24.12	24.65	24.21	24.15	23.75	24.21	23.99	24.25	24.26	24.24
H08		24.01	24.03	24.78	24.03	24.13	23.79	24.04	23.53	24.80	24.21	24.05
H09		24.05	24.00	24.04	24.03	24.19	23.73	24.13	23.53	23.64	24.93	24.04
H10		20.85	20.76	20.68		20.85	20.71	20.28	20.19	20.91	20.86	20.56
H11		20.88	20.77	21.04	20.78	21.06	20.85	20.23	20.52	20.89	20.94	20.64
H12		21.00	20.97	20.94	21.04	21.12	21.04	20.55	21.07	21.62	21.02	21.00

FIGURE 8-HKF

Position		NT Control Group	EDA Group 1	EDA+TAKi Group 2	EDA+P38i Group 3	EDA+ERKi Group 4	EDA+JNKi Group 5	III-1C Group 6	III-1C+TAKi Group 7	III-1C+P38i Group 8	III-1C+ERKi Group 9	III-1C+JNKi Group 10
A01	BCL6	29.65	30.16	29.71	29.25	30.42	29.90	28.76	29.01	29.25	29.71	29.60
A02	C3	34.42	34.78	35.25	33.85	35.60	33.50	34.79	37.65	35.12	37.00	
A03	C3AR1	33.91	34.51	34.75	34.72	34.29	34.48	35.70	37.09	34.72	36.39	34.96
A04	CCL11	34.34	34.75	35.63	35.09	35.20	35.03	32.85	34.18	34.01	33.69	34.17
A05	CCL13	35.80	37.21	36.58	36.71	36.60	37.02	37.08	38.36	37.24	37.82	38.45
A06	CCL16											
A07	CCL17	37.19	38.13	37.45	36.42	37.31	36.31	36.61	38.12	37.81	38.10	38.93
A08	CCL19	37.59	37.40	37.39	39.35	36.62	37.85	37.10	38.33	37.17	36.48	38.38
A09	CCL2	30.06	27.03	30.24	28.18	27.55	29.55	27.81	30.65	28.19	29.48	29.56
A10	CCL21	37.22	37.10	36.47	36.34	36.11	37.55	36.98	36.42	37.23	37.27	36.87
A11	CCL22									39.83		
A12	CCL23	39.66		39.09				37.25				
B01	CCL24		39.68			39.33	39.75					
B02	CCL3	38.76	38.79	38.71	38.37	38.15	37.50	38.71	38.08	38.39	39.09	37.50
B03	CCL4		38.48									
B04	CCL5	31.95	31.84	32.22	31.41	31.22	32.00	34.42	39.44	35.79	35.72	37.11
B05	CCL7	34.80	33.49	34.19	32.52	33.22	33.45	35.45	35.34	35.78	35.04	35.44
B06	CCL8	37.18	38.02	37.69	36.92	37.18	37.41	37.55	38.42	37.19	38.39	38.10
B07	CCR1	35.83	36.17	35.72	35.73	36.30	35.75	36.42	36.34	36.09	37.05	36.53
B08	CCR2	35.69	34.21	34.64	34.71	34.59	33.11	34.90	35.32	34.55	34.84	34.27
B09	CCR3	35.24	35.44	35.25	34.72	35.09	34.75	34.93	35.84	35.31	35.49	36.20
B10	CCR4	37.37	37.62	37.14	37.06	37.12	36.55	37.01	37.92	37.29	37.62	37.40
B11	CCR7	34.48	35.11	35.13	36.38	34.45	35.57	35.12	36.16	38.07	36.21	34.77
B12	CD14	33.19	33.71	33.80	33.11	33.58	33.65	34.05	34.21	34.03	34.96	33.75
C01	CD40	32.87	33.20	33.46	33.43	32.99	33.28	32.53	32.81	32.32	32.92	32.74
C02	CD40LG	35.07	34.89	35.30	34.82	34.55	37.29	34.63	34.49	35.44	34.96	35.48
C03	CEBPB	28.46	28.30	29.10	28.15	28.04	28.35	27.41	27.85	28.06	27.95	27.75
C04	CRP	39.65	39.82	39.25	39.16				39.24	39.57	39.94	
C05	CSF1	29.31	28.58	29.39	28.19	28.34	28.42	27.68	28.29	27.76	28.16	28.28
C06	CXCL1	28.83	24.41	27.97	24.37	24.89	25.65	25.20	29.00	25.62	26.86	27.23
C07	CXCL10	34.60	33.59	35.69	37.38	34.00	35.45				36.62	
C08	CXCL2	29.62	25.10	28.64	25.03	25.34	26.14	26.05	29.64	26.39	27.55	28.16
C09	CXCL3	32.19	26.58	29.02	26.14	26.25	27.31	28.10	30.20	27.93	29.07	29.63
C10	CXCL5	32.24	32.11	32.15	30.03	31.52	31.93	32.22	32.83	31.77	33.36	33.03

C11	CXCL6	26.66	26.46	26.86	26.42	26.46	26.50	27.79	29.33	28.52	29.21	29.42
C12	CXCL9	34.10	33.98	33.99	33.72	33.86	33.29	33.43	33.05	34.19	34.01	33.71
D01	CXCR1				39.94	39.79						
D02	CXCR2	32.15	32.12	32.49	31.94	31.90	31.86	31.89	32.43	32.19	32.70	33.09
D03	CXCR4	36.52	39.79	39.54		37.13					38.09	38.44
D04	FASLG	35.99	36.51	36.55	35.95	36.08	36.22	36.21	37.25	36.12	36.46	37.01
D05	FOS	31.18	31.16	33.41	28.92	34.08	33.08	30.86	32.12	30.74	32.88	31.29
D06	IFNG		37.22	37.24		36.17	36.56	37.81	38.36	37.56	37.26	37.55
D07	IL10	39.54	37.94	37.32	37.03	38.15	37.51	38.46	37.79	37.43	39.61	39.37
D08	IL10RB	28.02	28.11	28.36	27.79	27.72	28.10	27.57	27.52	27.51	27.85	27.60
D09	IL15	30.35	30.17	30.23	30.15	29.98	30.23	29.20	29.63	29.46	29.58	29.54
D10	IL17A						38.33			36.61		
D11	IL18	34.41	33.70	34.28	34.12	33.68	34.08	33.93	34.55	34.18	34.35	34.52
D12	IL1A	34.46	30.87	34.52	31.49	32.07	32.49	34.03	35.56	34.77	37.11	38.00
E01	IL1B	31.66	30.24	33.14	30.23	30.78	33.02	32.87	34.89	32.89	34.33	34.56
E02	IL1R1	28.16	28.41	28.45	28.20	28.19	28.04	26.69	27.59	27.32	27.73	27.31
E03	IL1RAP	30.12	30.18	30.10	29.70	30.03	29.89	28.61	28.98	28.97	29.30	29.10
E04	IL1RN	31.73	32.15	32.10	33.17	31.48	33.42	36.82	37.39	37.69	36.11	36.40
E05	IL22	34.26	34.51	34.28	34.57	34.02	34.79	34.38	34.22	34.30	34.30	33.92
E06	IL23A	33.67	33.41	34.06	34.20	33.59	33.91	33.50	33.51	33.97	33.95	34.24
E07	IL23R						38.89					
E08	IL5	37.66	37.39	36.54	37.24	35.33	35.70	38.41	37.68	38.34	36.07	36.58
E09	IL6	32.06	28.60	32.35	28.96	29.55	30.71	29.60	34.11	31.16	33.51	32.90
E10	IL6R	32.70	33.15	33.37	31.69	32.72	32.16	32.01	31.40	31.98	31.92	31.73
E11	CXCL8	29.00	24.43	28.60	23.67	25.15	26.32	26.85	31.29	26.66	28.73	29.91
E12	IL9	39.01	39.11	38.91		39.46		37.83	38.80	38.59	39.08	
F01	ITGB2	33.92	34.35	34.30	33.62	33.63	33.23	33.37	33.64	33.31	33.87	34.03
F02	KNG1			39.50								
F03	LTA	33.58	33.24	32.97	32.97	32.72	32.43	32.25	33.42	33.20	34.10	33.23
F04	LTB	33.95	33.85	33.80	33.38	37.54	33.17	33.23	34.38	33.58	34.26	34.19
F05	LY96	27.45	27.49	27.54	27.21	27.38	27.27	27.46	27.38	27.32	27.76	27.41
F06	MYD88	29.04	29.16	29.44	29.19	28.85	29.04	28.24	28.48	28.39	28.77	28.36
F07	NFKB1	30.20	29.34	30.43	28.88	29.09	29.43	28.72	29.14	28.60	29.17	28.95
F08	NOS2	39.32	37.21	38.83	36.04	36.88	37.39	38.65	36.72	37.45	38.00	38.05
F09	NR3C1	26.74	26.72	27.18	26.71	26.58	27.06	26.56	27.04	26.63	27.23	26.94
F10	PTGS2	30.59	28.33	31.15	28.61	28.96	30.08	31.12	31.69	31.27	31.89	31.44
F11	RIPK2	29.04	28.39	29.21	28.26	28.42	28.31	28.11	28.29	28.07	28.51	28.23

F12	SELE	36.51	33.60	36.31	32.87	34.19	36.40	36.80	36.31	34.54	37.42	37.51
G01	TIRAP	32.01	32.37	32.34	32.12	31.93	31.79	31.04	30.92	30.98	31.38	30.71
G02	TLR1	34.45	33.70	34.32	33.41	33.60	34.62	33.35	33.30	33.66	33.71	33.97
G03	TLR2	34.45	33.12	34.69	33.20	33.86	33.71	33.47	34.90	33.87	34.60	34.19
G04	TLR3	30.69	31.21	31.13	31.46	31.04	31.16	31.66	32.05	31.99	31.83	31.97
G05	TLR4	29.72	29.77	30.65	29.38	31.19	30.47	29.17	30.60	29.19	31.41	30.34
G06	TLR5	33.61	33.80	34.26	33.79	33.77	34.34	33.39	33.75	33.89	33.98	33.97
G07	TLR6	30.06	30.15	29.88	30.10	29.81	30.03	29.65	29.81	29.82	30.14	30.26
G08	TLR7	35.44	35.11	35.38	34.66	34.61	35.21	34.31	34.73	34.90	35.01	34.79
G09	TLR9	39.46		38.40				36.69		37.23	39.45	36.80
G10	TNF	38.22	32.15	39.43	31.53	31.71	33.13	34.35	36.97	34.32	34.55	35.81
G11	TNFSF14	37.35	37.07	35.97	38.06	36.79	36.36	38.14	37.30	39.74	37.66	36.95
G12	TOLLIP	28.30	28.62	28.59	28.25	28.32	28.24	27.19	27.21	27.50	27.56	27.36
H01	ACTB	20.00	20.14	20.44	19.90	19.87	20.00	18.65	18.73	18.61	19.12	18.52
H02	B2M	22.12	22.25	22.53	21.95	22.04	22.05	22.03	22.10	22.01	22.41	21.96
H03	GAPDH	20.65	20.76	20.88	20.39	20.36	20.47	19.98	20.24	20.11	20.46	20.09
H04	HPRT1	28.25	28.35	28.46	28.13	28.14	28.17	28.04	28.00	27.84	28.23	27.79
H05	RPLP0	19.99	19.92	20.13	19.81	19.76	19.70	19.52	19.42	19.31	19.71	19.28
H06							38.55		39.48			
H07		24.43	24.71	24.91	24.73	24.61	24.64	25.03	25.48	25.35	25.51	25.31
H08		24.34	24.46	24.91	24.37	24.33	24.49	24.83	25.40	25.22	25.55	25.12
H09		24.37	24.51	24.97	24.37	24.36	24.46	24.75	25.43	25.22	25.56	25.05
H10		20.82	20.93	20.99	20.70	20.55	21.03	20.74	20.67	20.59	21.05	20.34
H11		20.93	20.80	21.08	20.64	20.48	20.71	20.89	20.70	20.75	21.04	20.41
H12		21.04	21.10	21.14	21.01	20.79	21.04	21.02	20.96	21.00	21.26	20.58

FIGURE 9 - FnEDA

	Inhibition of FnEDA-Induced Genes														
	A1F					HDF					HKF				
	DMSO	TAK1	p38	ERK	JNK	DMSO	TAK1	p38	ERK	JNK	DMSO	TAK1	p38	ERK	JNK
CCL2	59.2	0.0	0.3	0.2	0.1	39.9	0.0	0.3	0.5	0.4	8.7	0.1	0.4	0.6	0.4
CCL5						10.3	0.1	1.0	0.6	0.0					
CCL7	18.5	0.0	0.6	0.3	0.1	13.8	0.1	0.9	0.3	0.3					
CCL11	18.5	0.0	0.0	0.1	0.0	5.3	0.1	0.4	0.9	0.0					
CXCL1	232.5	0.0	0.9	0.5	0.2	119.2	0.0	1.1	0.6	0.1	22.6	0.1	0.9	0.6	0.2
CXCL2	210.4	0.0	0.9	0.6	0.2	156.5	0.0	1.0	0.8	0.1	24.3	0.1	0.9	0.7	0.3
CXCL3	154.1	0.0	0.8	0.8	0.2	126.5	0.0	1.1	0.8	0.1	51.9	0.2	1.1	1.1	0.2
CXCL5	7.4	0.1	1.3	1.1	0.6										
CXCL6	29.0	0.0	0.8	0.6	0.4										
CXCL8	134.1	0.0	2.0	0.8	0.1	246.6	0.0	1.0	0.3	0.0	25.3	0.1	1.4	0.5	0.0
CXCL10	7.7	0.0	0.1	0.1	0.0	14.8	0.0	0.2	0.6	0.0					
IL1A											12.7	0.1	0.5	0.4	0.2
IL1B						7.3	0.0	2.0	0.3	4.1					
IL6	37.4	0.0	0.5	1.8	0.1	203.9	0.0	0.5	0.6	0.0	11.7	0.1	0.7	0.4	0.0
PTGS2	6.1	0.1	0.6	1.3	0.2						5.1	0.2	0.7	0.5	0.3
TNF	17.2	0.0	0.4	0.2	0.2	15.7	0.0	0.6	0.7	0.2	7.6	0.0	1.3	1.1	0.2

	Regulation of FnEDA-Induced Genes														
	A1F					HDF					HKF				
	C	TAK1	p38	ERK	JNK	C	TAK1	p38	ERK	JNK	C	TAK1	p38	ERK	JNK
CCL2															
CCL5															
CCL7															
CCL11															
CXCL1															
CXCL2															
CXCL3															
CXCL5															
CXCL6															
CXCL8															
CXCL10															
IL1A															
IL1B															
IL6															
PTGS2															
TNF															

FIGURE 9 - FnIII-1

	Inhibition of FnIII-1c-Induced Genes														
	A1F					HDF					HKF				
	DMSO	TAK1	p38	ERK	JNK	DMSO	TAK1	p38	ERK	JNK	DMSO	TAK1	p38	ERK	JNK
**CCL11**	10.8	0.1	0.2	0.3	0.1										
**CCL2**	40.1	0.0	0.7	0.8	0.3	36.0	0.0	0.2	0.6	0.0					
**CCL7**	11.7	0.0	0.4	0.5	0.1	9.2	0.1	0.9	0.3	0.1	11.6	0.2	1.1	0.6	0.3
**CXCL1**	167.6	0.0	1.0	0.6	0.1	104.4	0.0	1.5	0.6	0.0	8.4	0.1	0.7	0.4	0.2
**CXCL2**	143.7	0.0	1.1	0.8	0.2	139.6	0.0	1.2	0.7	0.1	8.1	0.1	0.8	0.4	0.2
**CXCL3**	90.2	0.0	1.2	0.9	0.3	105.3	0.0	1.7	0.7	0.1					
**CXCL6**	14.8	0.1	0.3	0.2	0.1										
**CXCL8**	104.2	0.0	1.5	0.8	0.0	213.3	0.0	1.3	0.3	0.0					
**CXCL10**						9.5	0.0	0.3	0.4	0.0					
**IL6**	17.9	0.1	1.2	2.0	0.1	87.7	0.0	0.9	0.6	0.0					
**PTGS2**	6.0	0.1	0.6	1.0	0.2	6.7	0.0	0.4	0.5	0.1					
**TNF**	10.7	0.0	1.3	1.1	0.4	10.8	0.0	0.9	0.7	0.3					

	Regulation of FnIII-1c-Induced Genes														
	A1F					HDF					HKF				
	C	TAK1	p38	ERK	JNK	C	TAK1	p38	ERK	JNK	C	TAK1	p38	ERK	JNK
**CCL2**															
**CCL7**															
**CCL11**															
**CXCL1**															
**CXCL2**															
**CXCL3**															
**CXCL6**															
**CXCL8**															
**CXCL10**															
**IL6**															
**PTGS2**															
**TNF**															
