# Supplementary material for: Induction of pro-inflammatory genes by fibronectin DAMPs in three fibroblast cell lines: Role of TAK1 and MAP kinases
Source: PLoS One. 2023 May 25;18(5):e0286390. doi: 10.1371/journal.pone.0286390 (PMC10212165; doi:10.1371/journal.pone.0286390)
Supplement: S1 Raw images — (PDF) [file pone.0286390.s002.pdf]

Figure2A

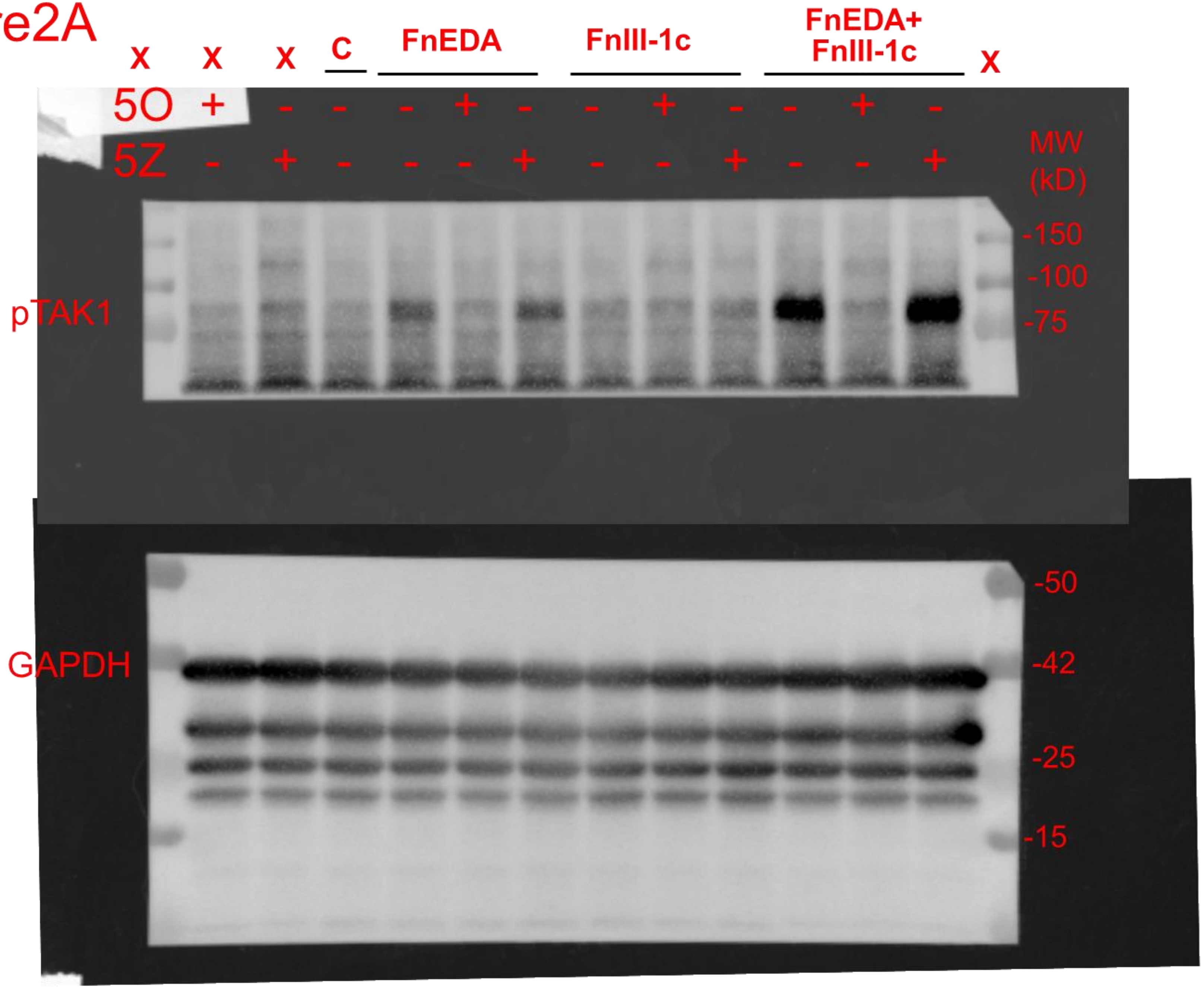

Captured using BioRad ChemiDoc MP Imaging System

Figure2B

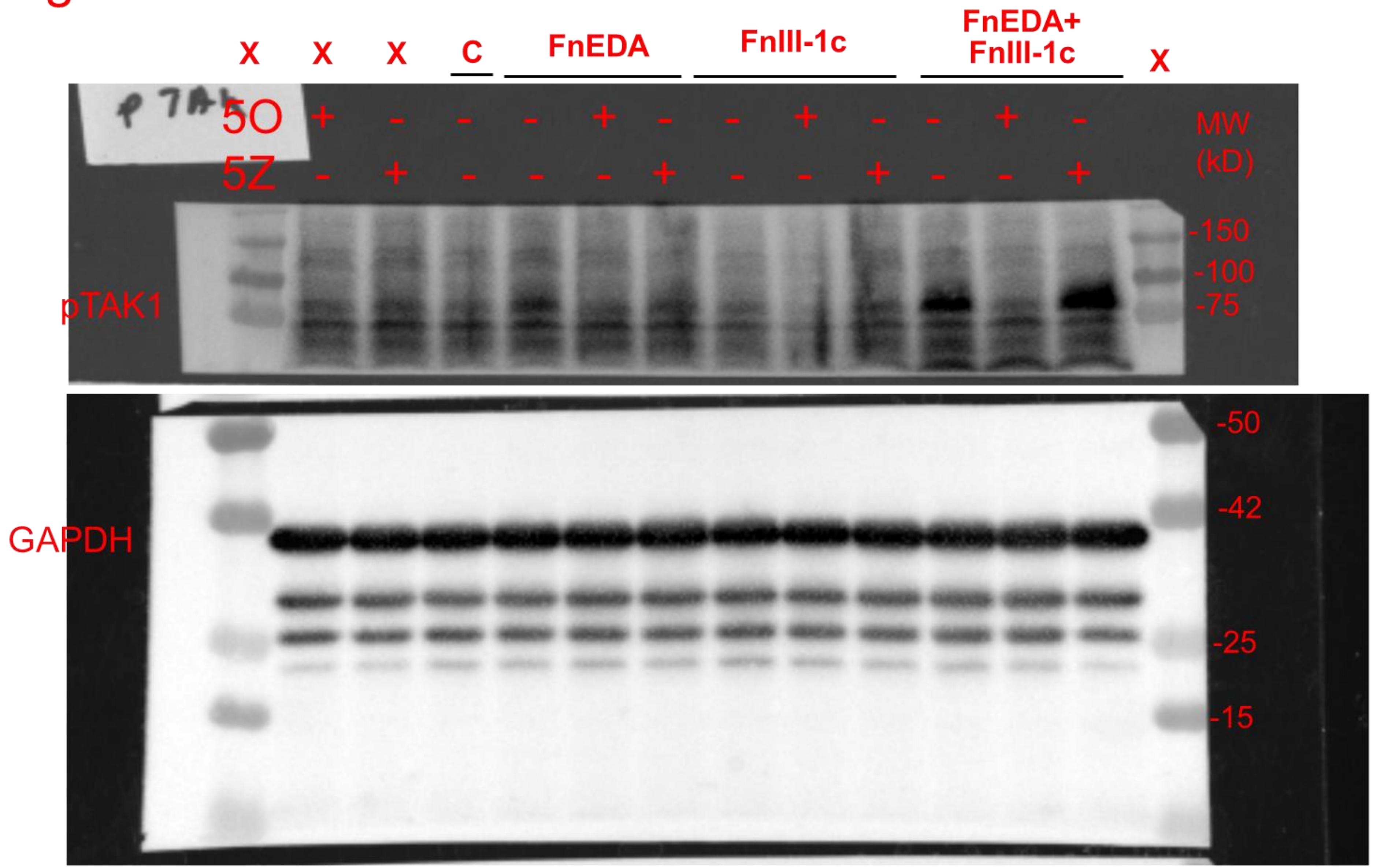

Captured using BioRad ChemiDoc MP Imaging System

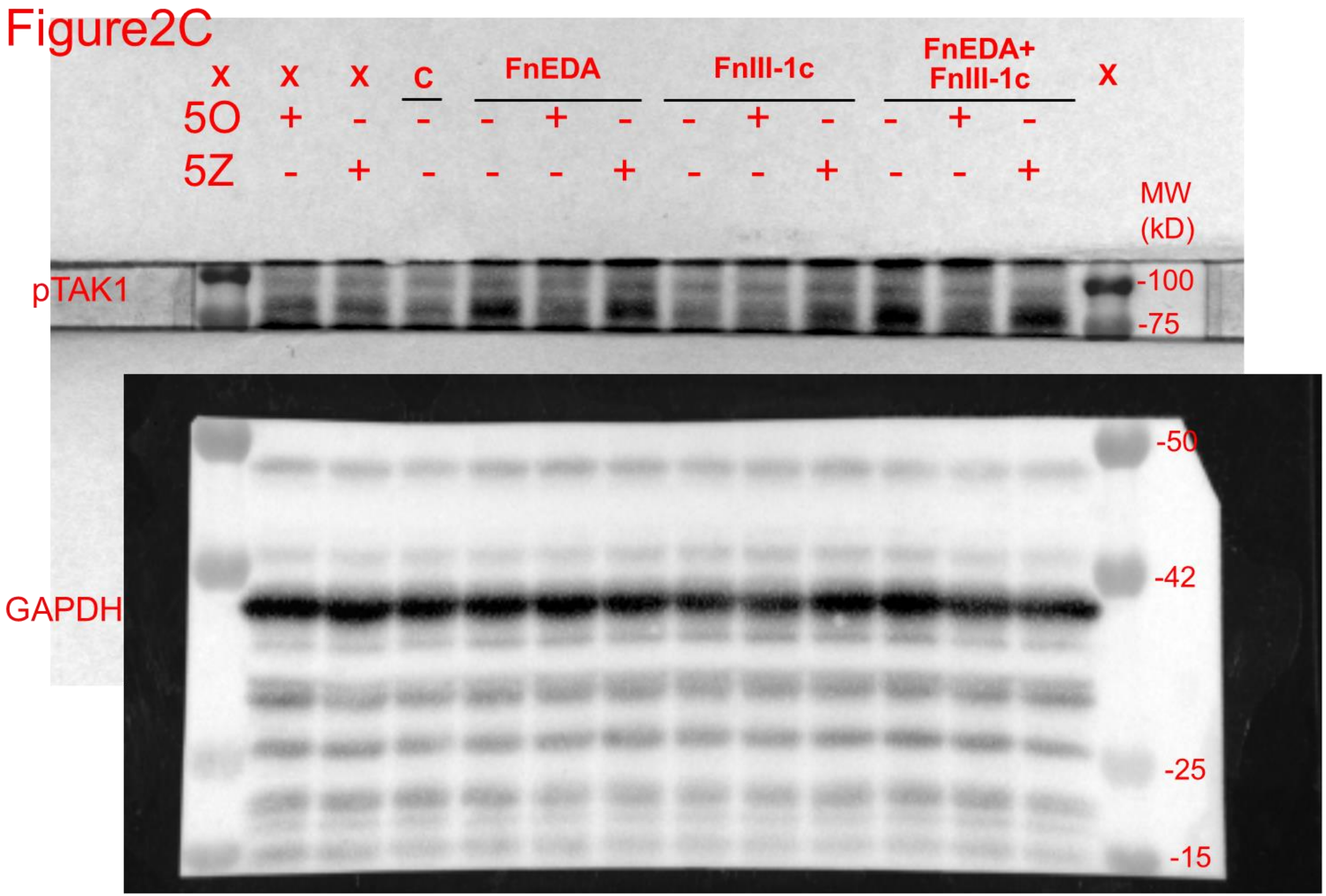

Captured using BioRad ChemiDoc MP Imaging System

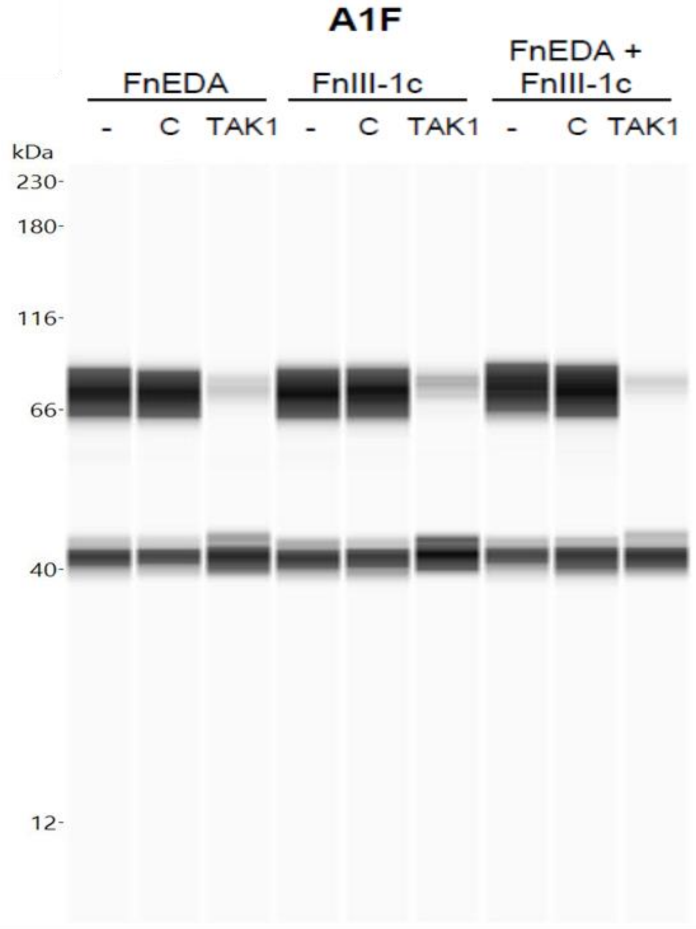

Captured on WES (ProteinSimple). Used to generate the top and bottom bands for Fig. 4A.

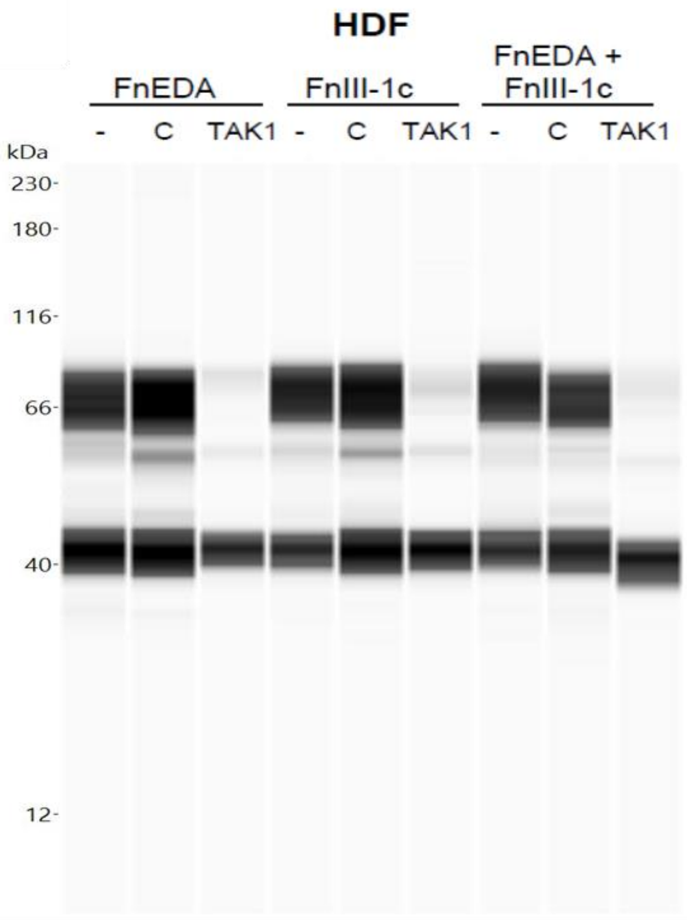

Captured on WES (ProteinSimple). Used to generate the top and bottom bands for Fig. 4B.

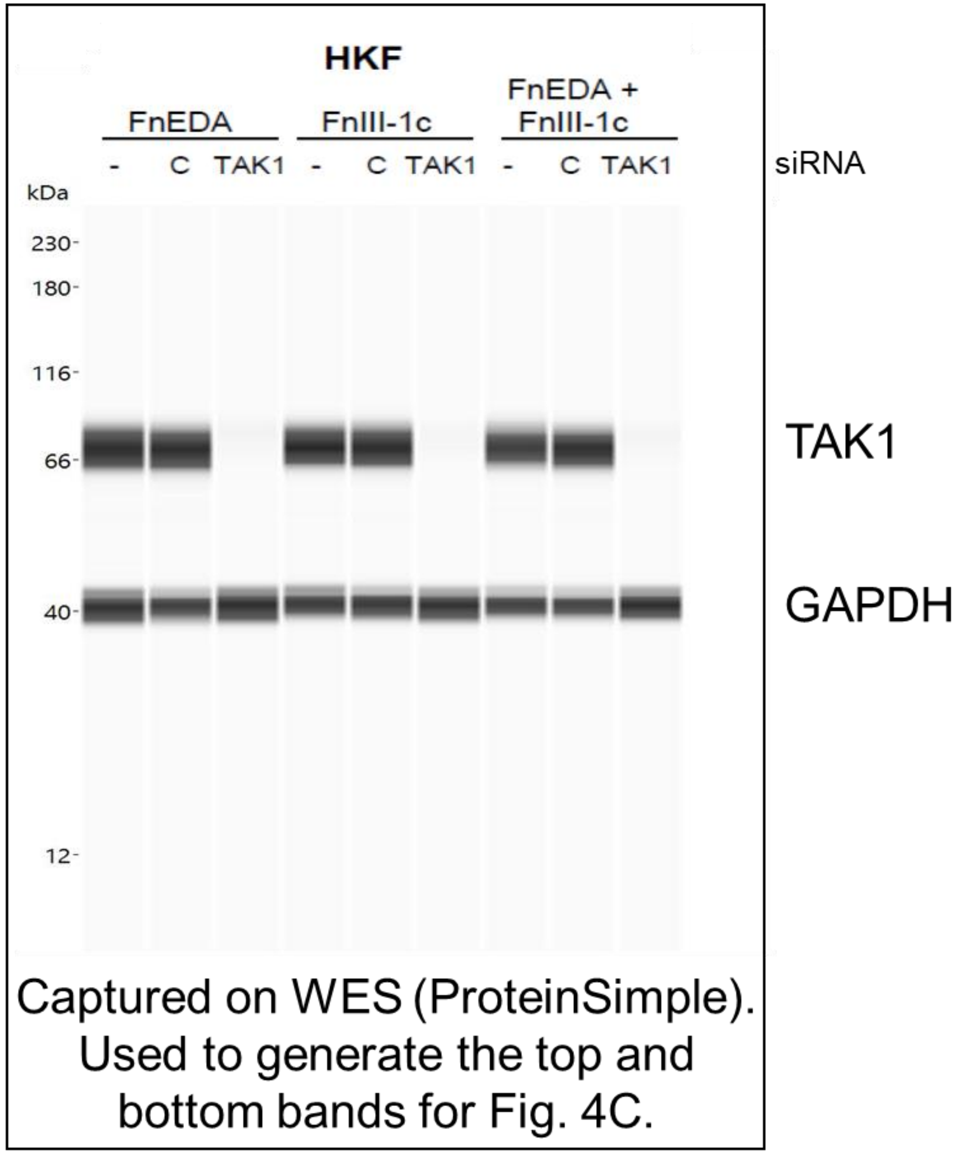

Captured on WES (ProteinSimple). Used to generate the top and bottom bands for Fig. 4C.

Figure 5A

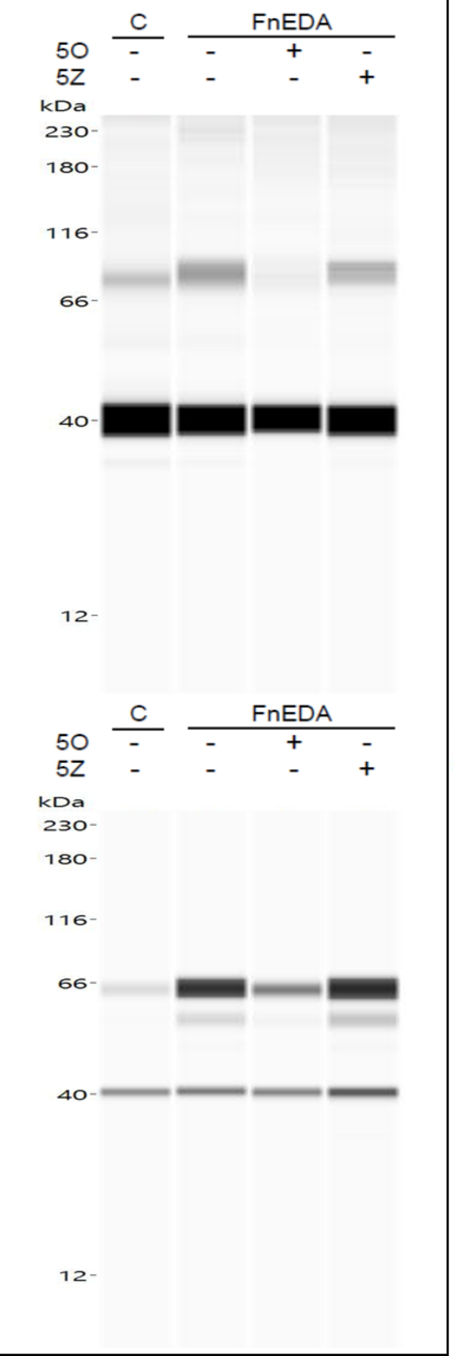

Figure 5B

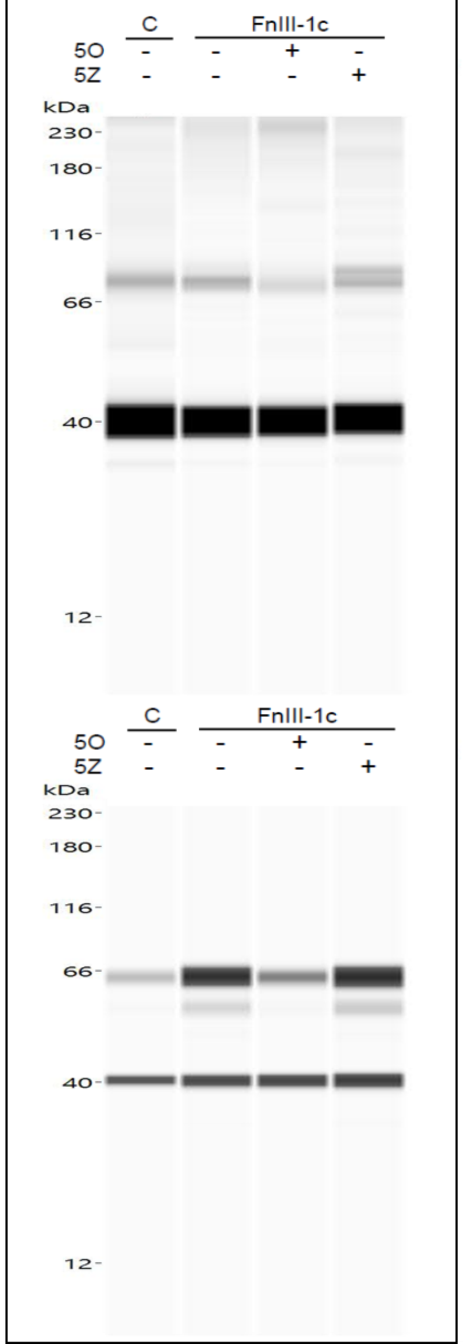

Figure 5C

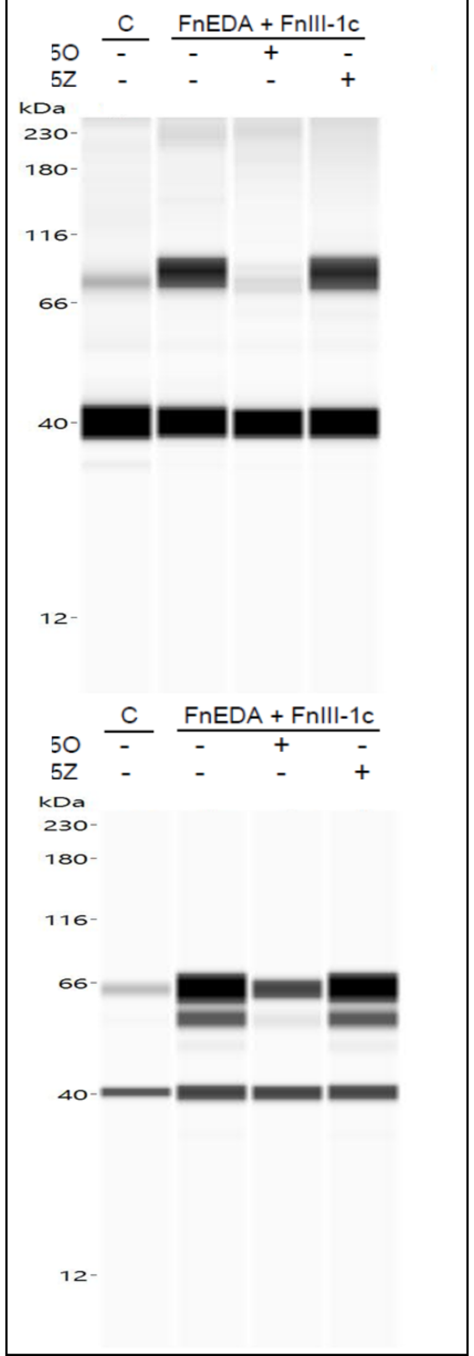

Captured on WES (ProteinSimple). Used to generate the top and bottom bands for Fig. 5A-C.

p-IKK

GAPDH

p-NF-κB

Captured on WES (ProteinSimple). Used to generate the middle band for Fig. 5A-C.

Figure 5F

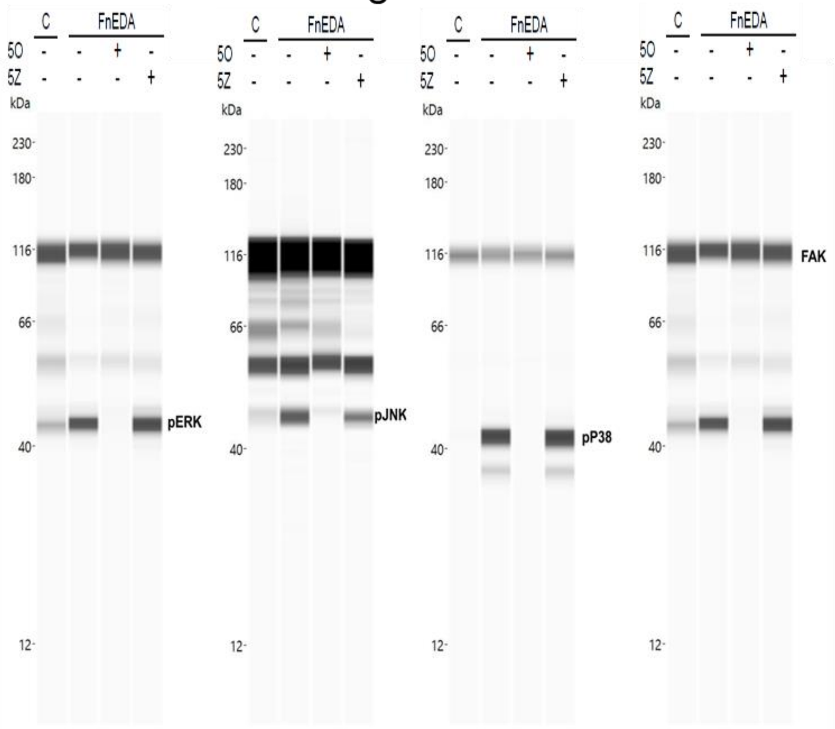

Captured on WES (ProteinSimple). Used to generate the top to bottom (Left→Right) bands for Fig. 5F.

Figure 5G

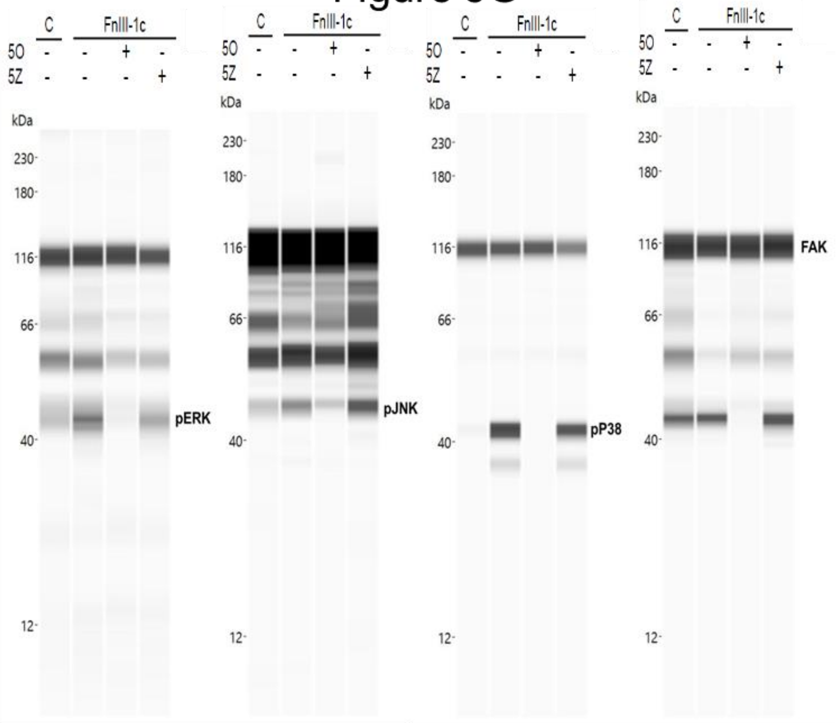

Captured on WES (ProteinSimple). Used to generate the top to bottom (Left→Right) bands for Fig. 5G.

Figure 5H

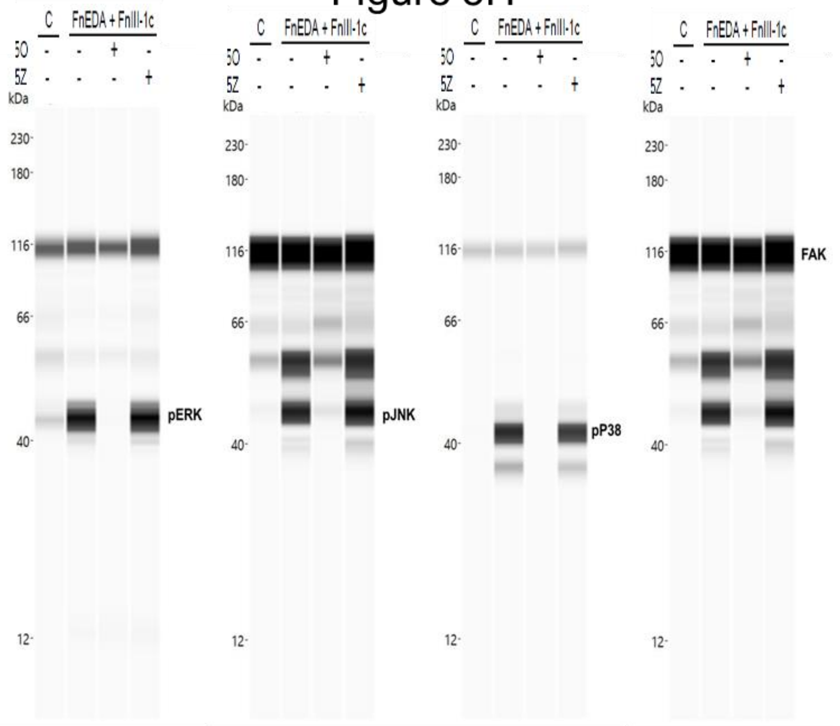

Captured on WES (ProteinSimple). Used to generate the top to bottom (Left→Right) bands for Fig. 5H.

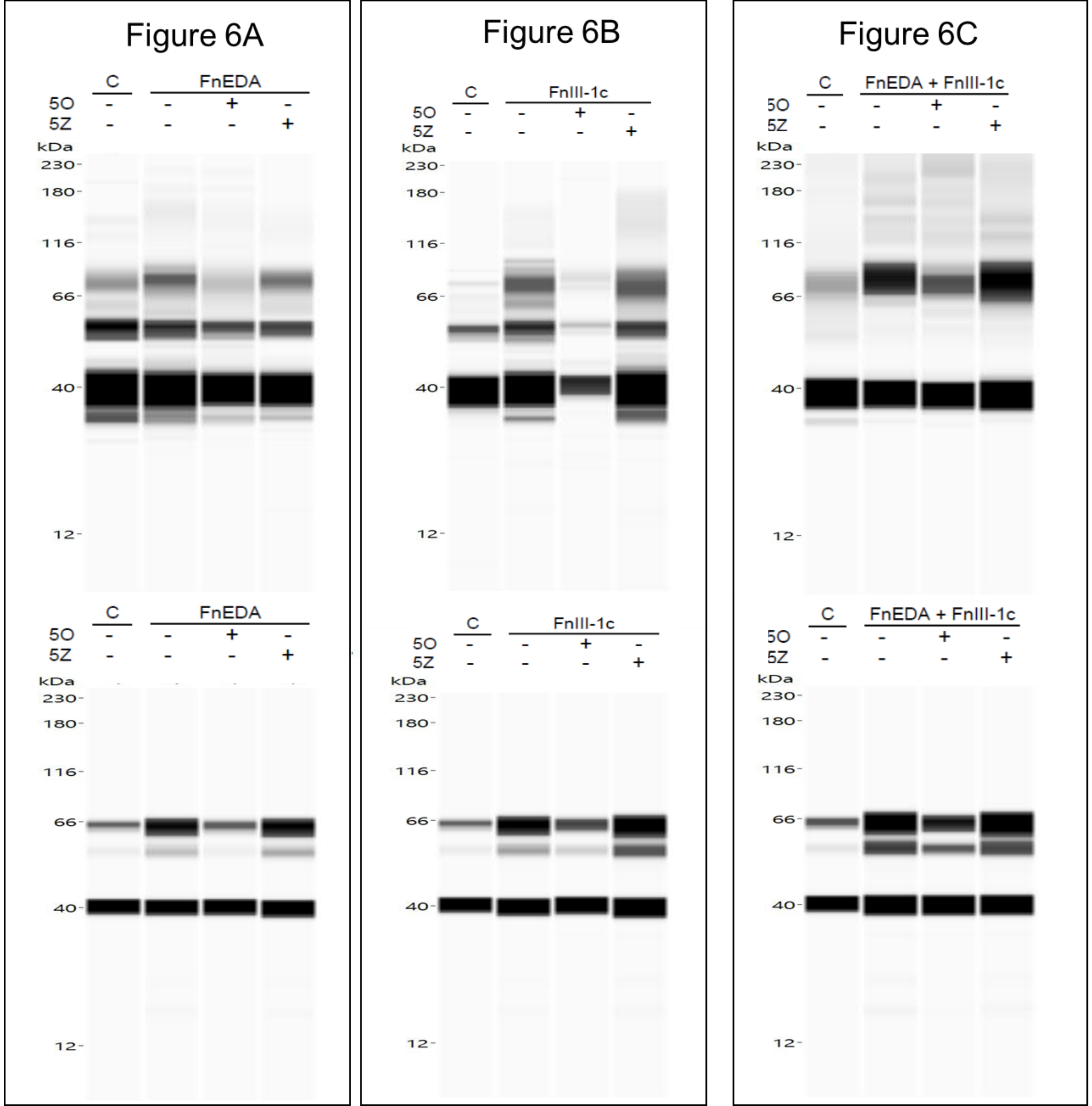

Captured on WES (ProteinSimple).
Used to generate the top band for Fig. 6A-C.

Captured on WES (ProteinSimple).
Used to generate the middle &bottom bands for Fig. 6A-C.

Figure 6F

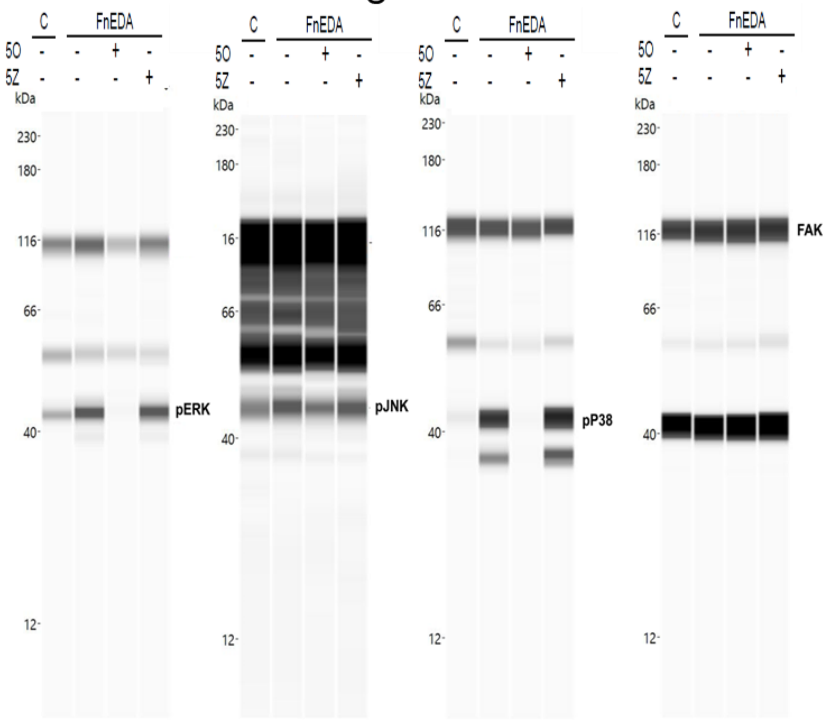

Captured on WES (ProteinSimple). Used to generate the top to bottom (Left→Right) bands for Fig. 6F.

Figure 6G

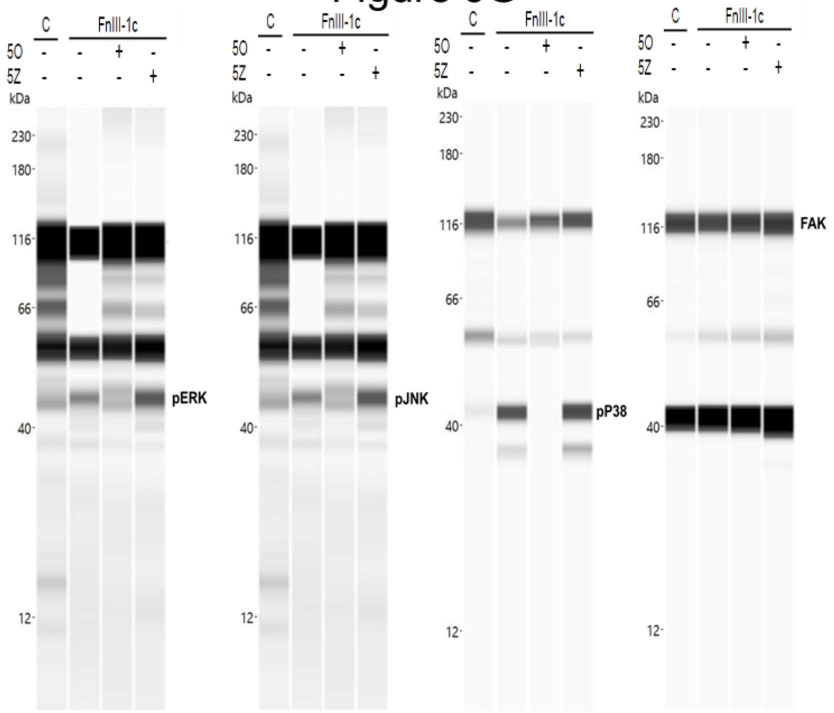

Captured on WES (ProteinSimple). Used to generate the top to bottom (Left→Right) bands for Fig. 6G.

Figure 6H

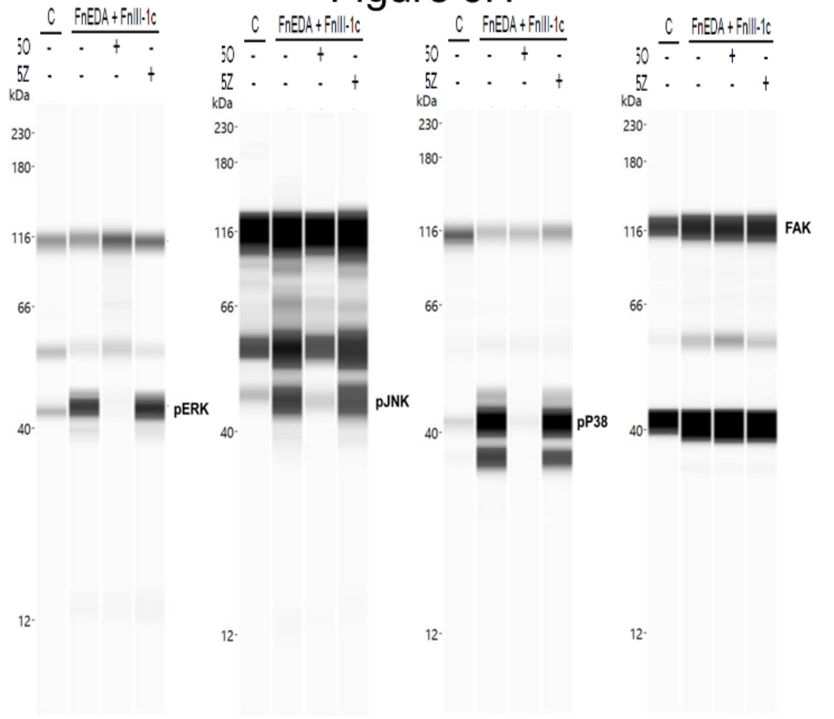

Captured on WES (ProteinSimple). Used to generate the top to bottom (Left→Right) bands for Fig. 6H.

Captured on WES (ProteinSimple). Used to generate the top band for Fig. 7A-C.

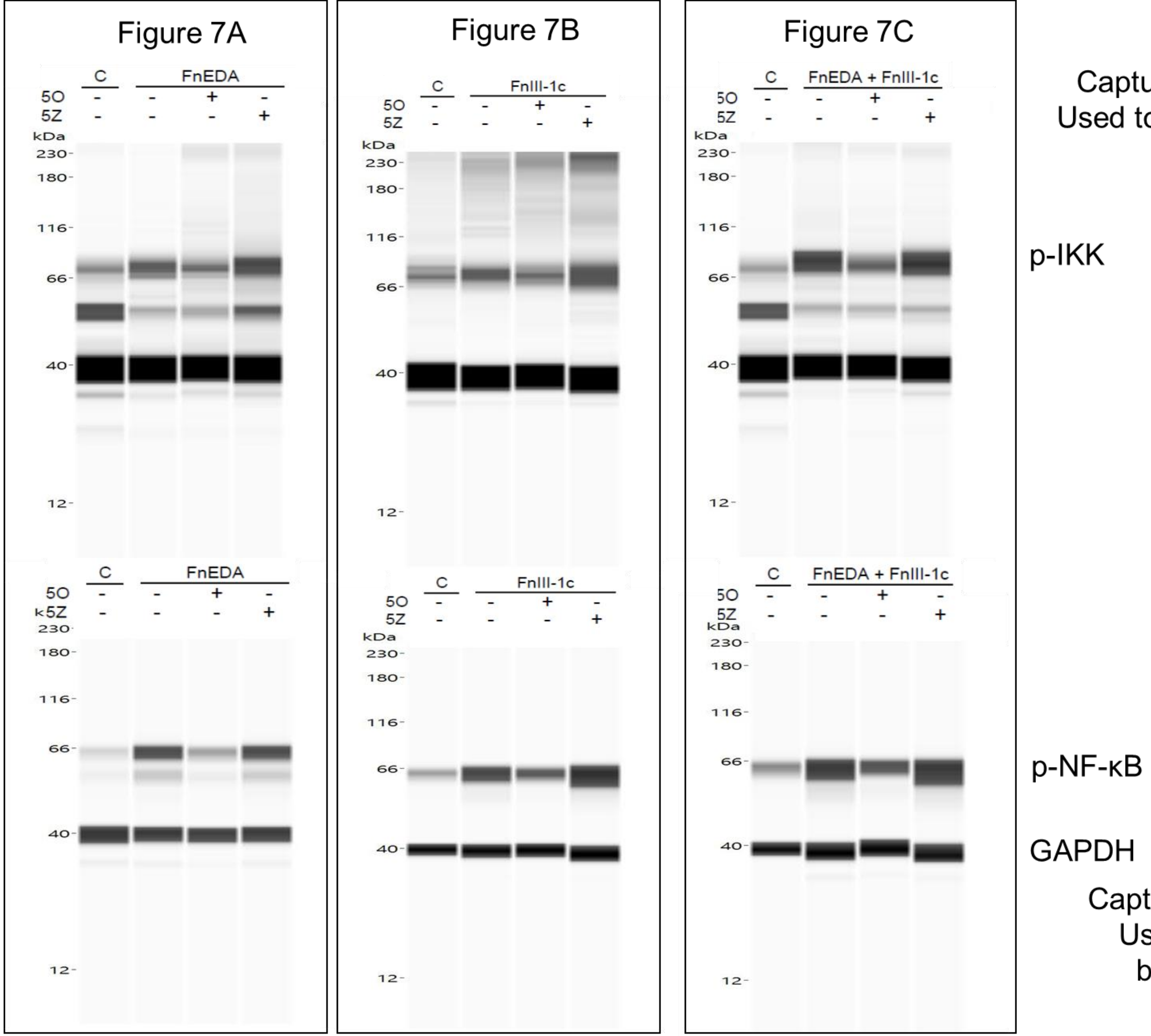

Captured on WES (ProteinSimple). Used to generate the middle & bottom bands for Fig. 7A-C.

Figure 7F

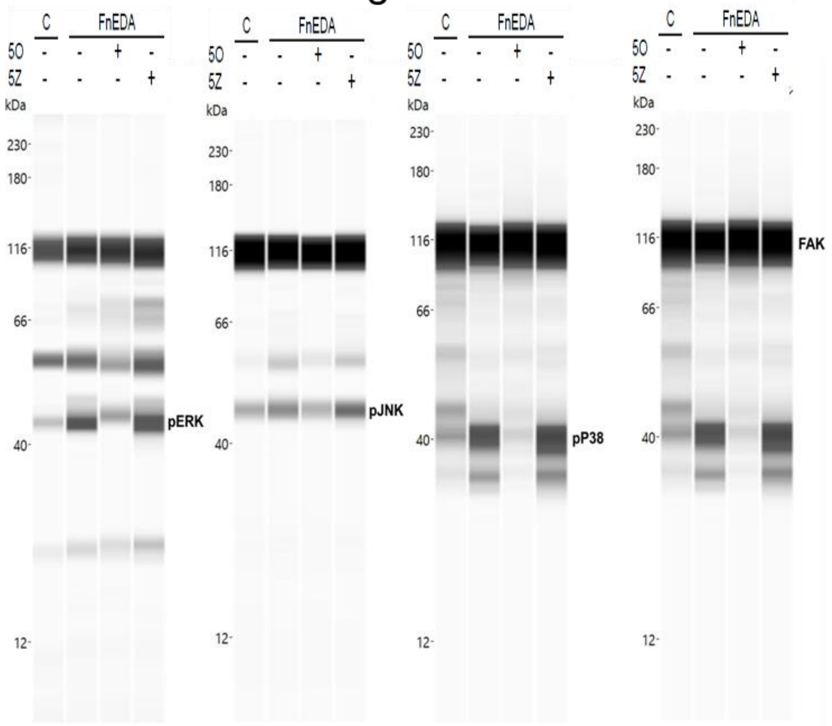

Captured on WES (ProteinSimple). Used to generate the top to bottom (Left→Right) bands for Fig. 7F.

Figure 7G

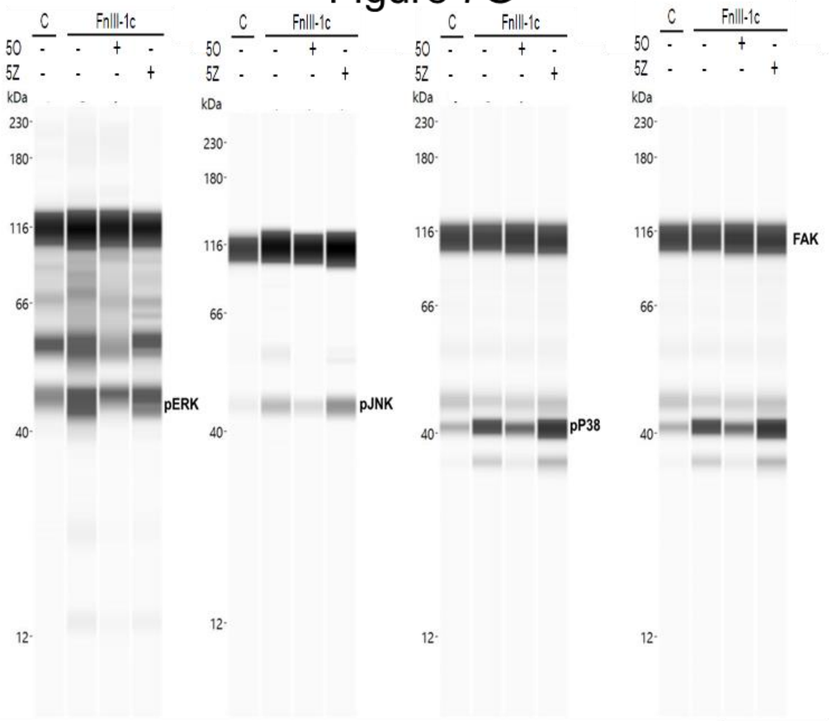

Captured on WES (ProteinSimple). Used to generate the top to bottom (Left→Right) bands for Fig. 7G.

Figure 7H

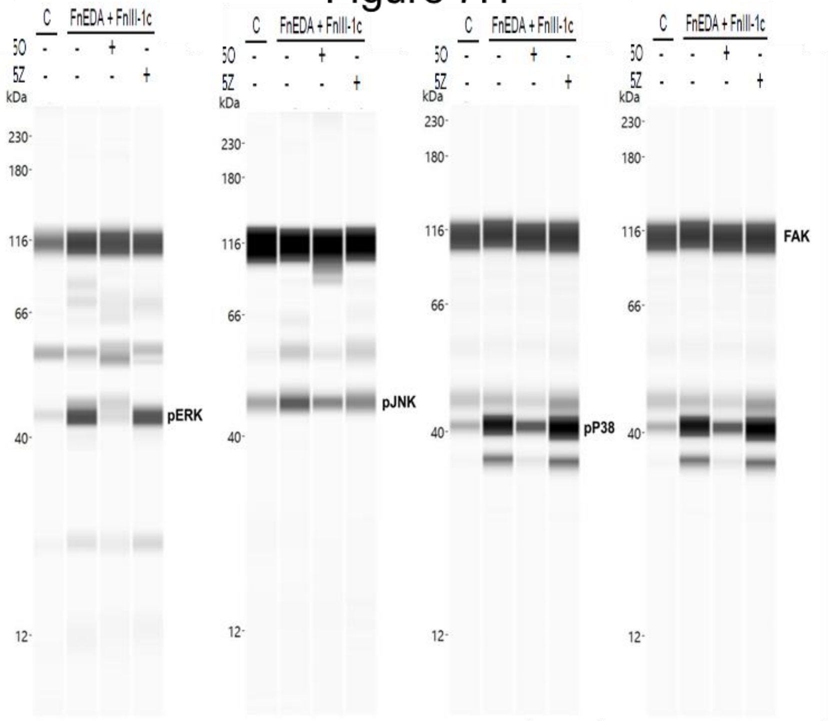

Captured on WES (ProteinSimple). Used to generate the top to bottom (Left→Right) bands for Fig. 7H.
