# Supplementary material for: Induction of pro-inflammatory genes by fibronectin DAMPs in three fibroblast cell lines: Role of TAK1 and MAP kinases
Source: PLoS One. 2023 May 25;18(5):e0286390. doi: 10.1371/journal.pone.0286390 (PMC10212165; doi:10.1371/journal.pone.0286390)
Supplement: Attachment — Submitted filename: Response to Reviewers.docx [file pone.0286390.s003.docx]

**Response to Reviewers**

Response to Reviewer 1:

There are no blot data in Figures 6F, 6G and 7F. *These data are now included.*

Response to reviewer 2

FnIII-1c appears to be a weaker DAMP than FnEDA and did not show a very robust response. Do the authors have any explanation why this would be the case?

*This is an interesting question.* *We do not know the underlying mechanism of how these DAMPs activate TLR signaling. Presumably activation of TLR4 by Fn DAMPs may involve ancillary proteins or adaptor molecules as have been described for LPS. Identification of such molecules and their role in regulating downstream signaling may provide further insight into this question.*
